# The performance of tranchet blows at the Late Middle Paleolithic site of Grotte de la Verpillière I (Saône-et-Loire, France)

**DOI:** 10.1371/journal.pone.0188990

**Published:** 2017-11-30

**Authors:** Jens Axel Frick, Klaus Herkert, Christian Thomas Hoyer, Harald Floss

**Affiliations:** 1 Department of Early Prehistory and Quaternary Ecology, Institute of Pre- and Protohistory and Medieval Archeology, Eberhard Karls University of Tübingen, Schloss Hohentübingen, Tübingen, Germany; 2 Projet collectif de recherche (PCR) "Le Paléolithique supérieur ancien en Bourgogne méridionale” associated with UMR 6298 ARTeHIS at the Université de Bourgogne, Dijon, France; 3 DFG CRC 1070 "RessourcenKulturen" B01, Tübingen, Germany; University at Buffalo - The State University of New York, UNITED STATES

## Abstract

This paper focuses on the technological characteristics of *Keilmesser* with a lateral tranchet blow modification on the cutting edge. It examines the underlying technological production of these bifacial objects: this implies the evaluation of their working stage succession, as well as produced forms necessary for the execution of tranchet blow performance. Furthermore, it offers a techno-morphological description of these enigmatic tools. The *Keilmesser* with tranchet blow and corresponding blanks of tranchet blows from Grotte de la Verpillière I in Germolles (Saône-et-Loire, France) are used as case study. The collection of *Keilmesser* with tranchet blow and corresponding blanks of tranchet blow has been massively expanded with new fieldwork and the review of ancient assemblages. The majority of the pieces were made on blanks from local raw material. The evaluation of the underlying production concept shows that a *Keilmesser* with tranchet blow, regardless of the wide range of morphologies and matrix size, always consists of specific parts that are necessary for the production sequence and the assumed function. The production of these pieces follows highly specific working stages, some of which can be interchanged in sequence. However, it is always the goal to obtain a low-angled cutting edge. The performance of a tranchet blow is not only an integral part of production, but it is rather the aim of the entire production.

## Introduction

In the course of Paleolithic research, the focus was placed primarily on retouched lithic objects. However, typological examinations of the pieces did not include a modification of a cutting edge that differed from orthogonal retouching.

The peculiarity of the cutting edge design of the artifacts discussed here was already recognized by Krukowski in the 1920s and 1930s at Ciemna cave, Poland [1]. However, it was not until the 1960s that a further site was discovered, which contained similarly modified stone artifacts, Buhlen, Germany [2]. Although the site of Balve was discovered before Buhlen, it was not discovered until the early 1990s that this modification of artifacts also existed here [3].

To date, only a few sites are known that contain modifications of lithic artifacts from a Late Middle Paleolithic context, which have been described as tranchet blow. So far, 14 sites with lithic objects with tranchet blow have been established (Table 1). The number of technological studies on *Keilmesser* with tranchet blow focusing on production sequences is extremely limited. In addition to this study, pieces of the Abri du Musée, Ciemna, Buhlen, Mont du Beuvry and the Grotte de la Verpillière II were studied in detail. It therefore seems necessary for us to proffer the pieces of this work as a further reference point, so that the at present very manageable quantity of *Keilmesser* with tranchet blow is duly incorporated into the research and can assume their important outstanding position within the production concepts of the Middle Paleolithic. The exact number of *Keilmesser* with tranchet blow from the Late Middle Paleolithic in Europe that have been found so far is very difficult to estimate. This is due to the fact that the tranchet blow modification has not been noticed by all researchers of the material and therefore the pieces have not been published as such.

**Table 1:** Sites with a Late Middle Paleolithic context that is considered to be evidenced and which show lithic objects with tranchet blow.

Site	Literature
Abri du Musée in Les Eyzies	[13, 14]
Albersdorf	[63]
Balve	[3]
Buhlen	[2, 32, 37]
Ciemna	[1, 64–68]
Grotte de la Verpillière I	[8, 17–21, 24]
Grotte de la Verpillière II	[11, 16, 17]
Hohler Stein near Schambach	[63]
Inden-Altdorf	[69]
La Baume de Gigny	[14, 70]
Mont du Beuvry near Béthune	[71]
Ramioulle	[72]
Sesselfelsgrotte	[49]
Villemaur-sur-Vanne	[55]

In addition, it is important to know that such modifications, if they were detected, were given various different names in the literature, (e.g. tranchet blow, *Schneidenschlag*, para-burin, *coup de tranchet*, and so on). The names found in the literature and the research history for this modification will be published elsewhere in the near future [4]. Similarly, tranchet blows were also interpreted as different burins (cf. [5]) or were not taken into account in the analyses. Only few typological works mentioned that this particular modification existed, but it was rarely included in the classification of the pieces. Thus, pieces with and without tranchet blow were given the same typological name [6–8].

This paper discusses technological aspects of asymmetrically (bifacially) backed knives with a lateral tranchet blow (TB) modification in a longitudinal manner on the cutting edge at Grotte de la Verpillière I. Also known as *Keilmesser*, Pradniks or *Faustkeilschaber* [9–11], these tools are primarily present in a Late Middle Paleolithic context of Europe [12]. The term *Keilmesser* is given preference here.

The tranchet blow [11, 13–17] modification creates a low angled, sharp and stable cutting edge. In combination, the resulting technologically unique tools are named *Keilmesser* with tranchet blow (KMTBs).

Newly performed excavations at Grotte de la Verpillière I (VP I) and the compilation of all trackable finds from ancient excavations [18–21] now allow an extended analysis of these finds.

An overview discussing the majority of bifacial objects from both Grottes de la Verpillière I & II (VP I & VP II) until 2014 was recently given elsewhere [17], based on first observations from Floss [20] and Frick [21].

This paper aims to give a techno-morphological definition of these tools and discusses their entire production process, which is entirely targeted to produce specific shapes of surfaces and edges necessary to enable the performance of the cutting-edge formation with a tranchet blow along the intended cutting edge. The entire KMTB concept shows a high flexibility. It can be performed on different matrices and the individual working stages are often interchangeable.

In addition, a tranchet blow modification can be used to maintain a dull cutting edge.

### Grotte de la Verpillière I

**Fig 1:**
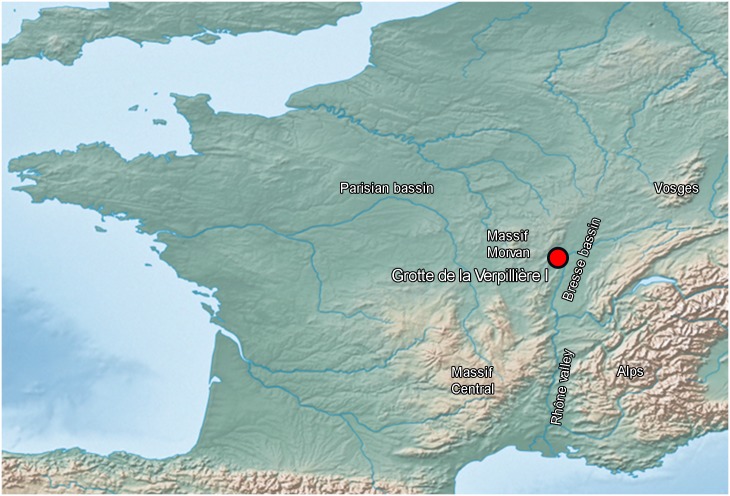
Location of Grotte de la Verpillière I (white point with black fringe). Position of the site on a relief map of Western Europe (base map from Natural Earth. Free vector and raster map data @ naturalearthdata.com).

The site of Grotte de la Verpillière I is situated in the small village Germolles (Mellecey commune) in the Saône-et-Loire department in Eastern France (Fig 1). The site is situated on the eastern cliff face of the Montadiot massif (around UTM 31 O 633000 N 5185500; 212 m a.s.l.) in the small valley of the Orbize River. The site is named after the local sub-district of Verpillière and was formed by erosion of soft limestone elements of the Upper Oxfordian formation. It is actually a rock shelter sealed by collapses at the opening, rather than a cave, as its name suggests.

It has been known as an archeological site since 1868, when it was discovered during road construction. In the same year, Ch. Méray and collaborators conducted first excavations [22, 23]. Since that time, around 20 excavations have taken place in the rock shelter and on its terrace [18, 19]. The site is not only well known for its assemblages of *Keilmesser* from the Middle Paleolithic [8, 24], but also for its contribution to establish the Aurignacian [25]. It has also yielded the easternmost known Châtelperron points [20, 26]. Recent excavations (2006 to 2016) unearthed artifacts attributed to the Late Middle Paleolithic, Châtelperronian, Aurignacian and Gravettian, and, to a much lesser extent, Neolithic and Medieval times.

### *Keilmesser* with tranchet blow from the site

**Fig 2:**
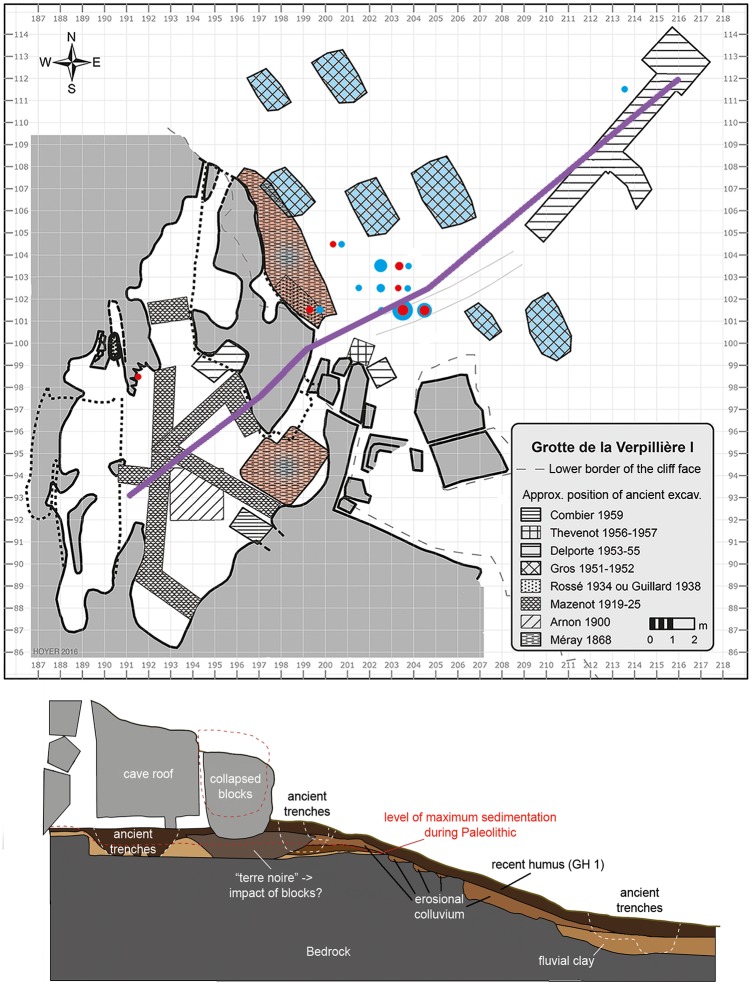
Spatial distribution of *Keilmesser* with tranchet blow and blanks of tranchet blows at Grotte de la Verpillière I. a) Plan view of the rock shelter with evaluated positions of ancient trenches and exact positions of the new excavations (base data from cave floor plan measurements by Beutelspacher, Floss, Jantschke & Woerz, August 2–3, 2003, data use with permission of Floss). Blue shades or dots signal the position of blanks of tranchet blows and red shades or dots signal the position of KMTBs. Sharply delineated symbols represent objects from the Floss excavation. The amount is represented in the size of the symbol per square meter. Shaded areas represent objects from ancient excavations (and maybe surface collections) without exact localization. The exact quantity per activity is displayed in Table 1. The violet line indicates the position of the cross section shown below. b) Cross section of the rock shelter, collapsed rocks and the hillside in front of the rock shelter (GIS and three-dimensional reconstruction of both views: Hoyer).

Systematic research about lithic objects with TB modification and corresponding blanks of TBs were able to evaluate n = 99 objects. To date, the assemblage from VP I contains n = 44 KMTBs (Table 2) and n = 55 blanks of TBs (Table 3) from different collections and excavation activities. As early as 1868 (first excavation of Méray) it was recognized that these pieces were special. Subsequently n = 3 KMTBs where depicted in the excavation report in 1876 [23]. One hundred years later, n = 7 KMTBs from the Méray excavation were a subject of study for correlations with similar finds from Poland and Germany [8]. The resumption of research took place in 2005 by Floss in reviewing the material from VP I in ancient collections [20]. The newly performed excavations could significantly increase the amount of KMTBs and blanks of TBs (the spatial position of the pieces is displayed in Fig 2).

**Table 2:** Assemblages of *Keilmesser* with tranchet blow from Grotte de la Verpillière I.

Collection	Activity	Year^c)^	Number
Méray (CA 27^a)^, archived at Musée Denon in Chalon-sur-Saône)	Méray excavation	1868	4
Jeunet (81.12.1^b)^, archived at Musée Denon)	Méray excavation	1868	5
Jeannin (archived at the University of Tübingen)	Méray excavation	1868	9
Floss (archived at the University of Tübingen)	Floss excavation	2011, 2015 and 2016	26
Total			44

Four different collections from the site contain *Keilmesser* with tranchet blow.

^a)^ Inventory number at Musée Denon

^b)^ Inventory number at Musée Denon

^c)^ Year of excavation

**Table 3:** Assemblages of blanks of tranchet blows from Grotte de la Verpillière I.

Collection	Activity	Year^c)^	Number
Jeannin (currently archived at the University of Tübingen)	Méray excavation	1868	1
Pelatin (currently archived at the University of Tübingen)	Surface collection and excavation	1970s	4
Aimé (89.78.1^a)^, archived at Musée Denon)	Surface collection and excavation	1970s	1
Gros (02.14^b)^, archived at Musée Denon)	Surface collection and excavation	1950s	3
Floss (archived at the University of Tübingen)	Floss excavation	2015 and 2016	46
Total			55

Five different collections from the site contain blanks of tranchet blows.

^a)^ Inventory number at Musée Denon

^b)^ Inventory number at Musée Denon

^c)^ Year of excavation

KMTBs are known from two activities on the site. On the one hand, they are known from the first excavation at the site in 1868, stored in different collections (Méray collection n = 4; Jeannin collection n = 9 and Jeunet collection n = 5). On the other hand, recent fieldwork (2006–2016) by Floss was able to unearth another n = 26 of these remarkable tools. With the exception of one KMTB from the interior of the rock shelter (found 2011 in a sediment unit containing a mixture of Middle and Early Upper Paleolithic items) all other KMTBs from the new excavations were situated directly in front of the former rock shelter in colluvial sediment units (see Fig 2). The Méray excavation in the 19th century was also situated in the recent entrance and in front of the collapsed rock shelter [18, 19].

It can therefore be assumed that the deposition of the pieces and their corresponding blanks occurred in the forecourt and entrance area of the rock shelter during the Middle Paleolithic, marking the approximate position of the pieces left behind.

It is noticeable that the pieces from the old excavations are relatively large compared to the pieces from the more recent fieldwork. This is not particularly surprising, but a phenomenon that is probably known from many sites. Due to the rough excavation methods of early research, small pieces were only rarely detected. Due to the intensive water screening and sorting work in modern fieldwork, we are in the fortunate position of being able to recover even tiny artifacts. Likewise, most of the new pieces come from areas already excavated in the 19th century. This circumstance also explains that most of the small blanks of TBs described below originate from the new fieldwork and were not found in the ancient excavations.

Detailed data are currently available for n = 43 KMTBs; a further KMTB could not be analyzed in detail and was therefore excluded from the following studies.

### Corresponding blanks of tranchet blows

**Fig 3:**
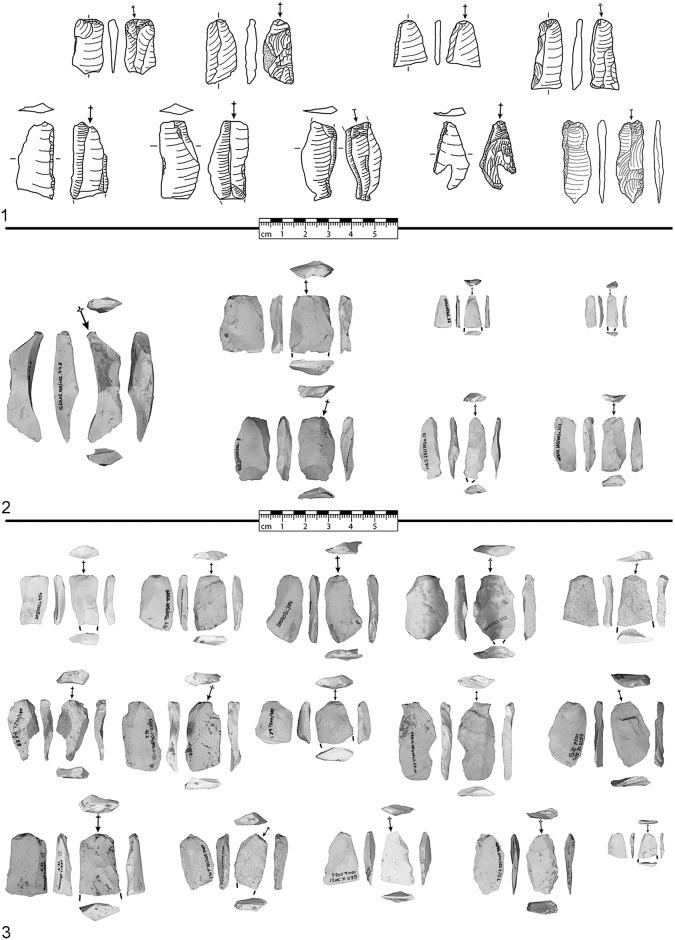
Selection of blanks of tranchet blows from Grotte de la Verpillière I. 1) Drawing from ancient collections; 2) Photographic image from the 2015 excavation and 3) Photographic image from the 2016 excavation. Arrows signal blow technique (spherical end = direct-hard blow, circle end = direct-soft end, without spherical or circle end = basal part is missing and the blow technique cannot be determined).

To date, n = 55 blanks of TBs are known (see Table 3, a selection is displayed in Fig 3). One blank of TB is present from the Jeannin collection (Méray excavation in 1868, red fields with dashed line in Fig 2a). However, this piece was not recognized and thus not published by the early researchers.

There are n = 3 blanks of TBs present from surface collections and test pits by A.-Ch. Gros from the 1950s (blue fields with cross-hatching in Fig 2a), n = 1 derives from the Aimé collection (excavation and/or surface collections from the 1970s) and n = 4 from the Pelatin collection (excavation and/or surface collections from the 1970s).

New fieldwork could unearth another n = 46 blanks of TBs from the front of the rock shelter (n = 11 from 2015 and another n = 35 from 2016). The evaluated distribution of the blanks of TBs is slightly different to them of the KMTBs, because the material from the new fieldwork was exclusively found in front of the rock shelter (see Fig 2).

Since most of the sediments underneath the rock shelter have been recovered during the course of the fieldwork, it is now unlikely at this point in time that further pieces can be recovered. On the forecourt, however, there are still several cubic meters of sediments, which could contain further pieces.

### Chronological fixation

None of the KMTBs originate from well stratified sediment layers, but there is some evidence for chronological fixation of these finds in the Late Middle Paleolithic context around the end of MIS 4 or the beginning of MIS 3. On the one hand, two AMS ^14^C samples on ivory from GH 15 (a mixed layer of Middle and Early Upper Paleolithic material) produced dates of >46.7 ka BP (OxA-32231) and >46.8 ka BP (OxA-32233). Both indicate that the under-laying GH 16 (probably the remains of an occupation floor inside the rock shelter, see also [27, 28]), has an *ante quem* date of older than 46 ka [26]. On the other hand, another line of evidence derives from Grotte de la Verpillière II (VP II), a site close-by with well stratified sediment layers of the Late Middle Paleolithic also containing bifacial objects and KMTBs [11, 16, 17]. There, dating attempts using various methods (IRSL, ESR-U/Th and AMS ^14^C) provided data indicating that all Middle Paleolithic layers (GH 3, GH 4x and GH 4) present there must be placed in the early MIS 3 between 40 and 50 ka (all of them contained KMTBs or blanks of TBs). In addition to these dating attempts, there is also chronological data from a *Keilmesser*-bearing site in the Department of Yonne (Le Dessous de Bailly in Champlost, excavated 1981–1992, directed by Girard and Farizy) with a tentative ESR date between 45 and 65 ka [29–31]. All of these dates have a large and tendentious character for the age of the KMTBs from VP I. However, they do make it likely that the actual age is situated in a MIS 4 to MIS 3 context.

## Techno-morphological definition of *Keilmesser* with tranchet blow

To reduce confusion about the naming of this tool (the literature provides numerous suggested names, e.g., *Pradnik*, *Prądnik*, *Prondnik*, *Prondnick*, *Prondtnick Prodnik*, *Keilmesser*, *Faustkeilmesser* or *Faustkeilschaber* and similar terms in other languages), we suggest the use of the term *Keilmesser* as a synonym for the term ‘asymmetrically backed knife’, which can be unifacially or bifacially worked. The term can be used with (KMTB) or without (KM) the addition of the term ‘lateral tranchet blow modification on the cutting edge’ (tranchet blow or TB).

**Fig 4:**
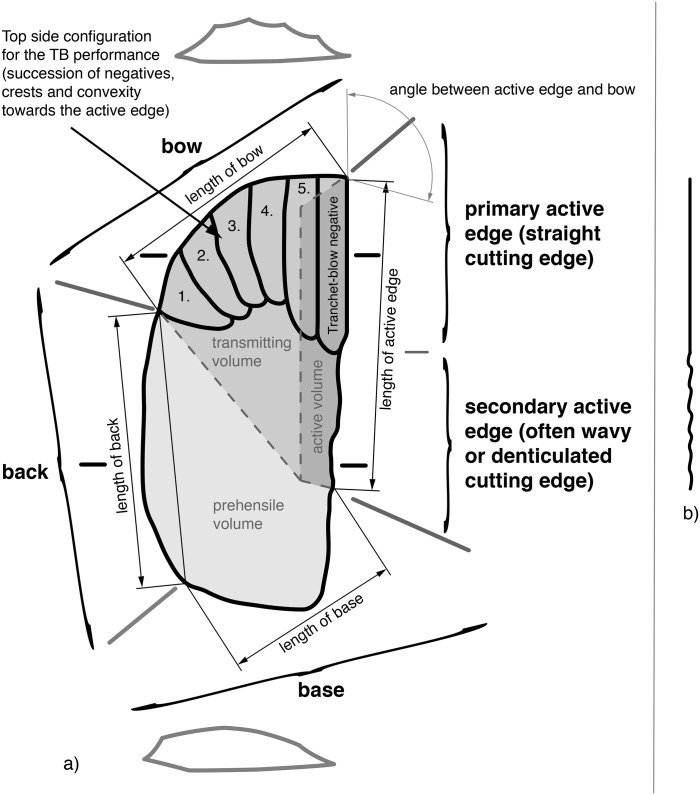
Schematic illustration of a *Keilmesser* with tranchet blow. a) Partition of a KMTB into different volumes (active, transmitting and prehensile), measurement positions of active edge, bow, back and base, as well as cross sections; b) Lateral view on the active edge showing its bipartition.

Techno-morphologically, KMTBs are described as follows: A *Keilmesser* with tranchet blow possesses a lithic volume. Its circumferential edge is divided into four sections (as viewed on the top side): one (at least) cutting edge, a back, a bow and a base (cf. [1, 7, 32], see Fig 4a). The prevalent cutting edge (a.k.a. active edge) is assumed to be used for different cutting directions [9, 33], mostly longitudinal cutting-in (<35°, for modes such as piercing, slicing or stabbing) and in addition, transversal cutting-off (> 35°, for modes such as scraping, whittling or smoothing). The intermediate angle allows both [34, 35]. This edge can be formed using retouch (retouch negatives are oriented orthogonally to the edge) and TB techniques (negatives of the TB are oriented along the edge).

Before a TB is performed, the prospective cutting edge is worked mostly bifacially in a manner that is comparable to the alternating unidirectional edge regularization as described by Bosinski [6]. The edge is not shaped alternately on both sides (a method used for roughing-out or double-symmetrical bifaces), but each side of the edge is formed in one go. This is the essential formation process for shaping surfaces and edges on these tools and can be described using turning and rotation during production, as preliminarily described by Weißmüller [36], see also [11].

Normally, the subsequent negative of the TB is situated laterally on the more convex surface (top side) of the object (Fig 4a). Afterwards, the cutting edge can be regularized using unifacial or bifacial retouch techniques. In many cases, the active edge is separated into two sections (if viewed from the lateral side), a straight one with the negative of the TB and a wavy or denticulated one (see Fig 4b).

On the opposite side of the cutting edge (on the other lateral side) there is a back, which can be natural (e.g., cortex or surface from preceding blank production) or shaped. The back continues into the terminally situated bow, which consists of a truncation as striking platform and convex surface modification for guiding the TB. Usually (as the term suggests), the bow is of arc-shape (as defined for *Pradnikmesser* by Bosinski [6]), but can also be straight in shape (*Klausennischemesser*, *Balver Keilmesser or Buhlener Keilmesser*, see also Jöris [10]). The review of the literature revealed different defined types (shape varieties) that can possess negatives of TBs [3, 9, 10, 32, 37]. Crucially, a TB can only be performed if a truncation (intentional or natural), a convexity (also intentional or natural) and a straight active edge (if viewed from the top, where the TB is anticipated) are present.

### Techno-functional units

In a techno-functional approach ([38, 39]) a KMTB consists of the volume at the back and the base as handle for the hand’s palm and the thumb (prehensile part), a volume represented at the bow on which a finger (often the index finger) can also press (transmitting part) and the active edge volume (transformative part) that provides the cutting edge (Fig 4a).

KMs, as well as KMTBs are prevalently interpreted as being hand-held and not fixed in a haft [9, 10, 32]. This was first proposed by Wetzel [40] for *Bocksteinmesser*. However, the resin finds (made from birch pitch) from Königsaue [41–43] indicate that bifacially backed objects could have been hafted as well, but such organic material is rarely preserved from the Middle Paleolithic. However, it is probable that *Keilmesser* can be either used hand-held or fixed in a haft.

### Laterality

**Fig 5:**
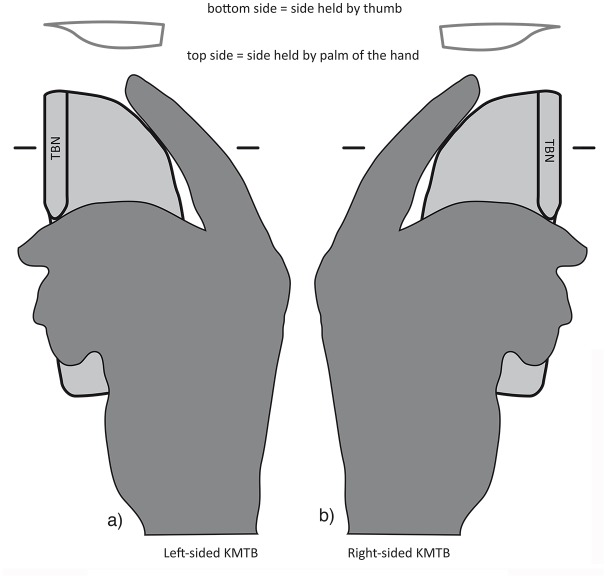
Handling of a *Keilmesser* with tranchet blow based on approaches of different scholars [13, 32, 33, 40, 60–62]. a) Handling of a left-sided KMTB and b) Handling of a right-sided KMTB.

The difference in shape of the top side (more convex surface) and bottom side (flatter surface) of KMs and KMTBs supplies evidence of laterality (Fig 5), if the tool is hand-held and not fixed in a haft. In such an approach, the tool is griped in such a way that the more convex surface and the back lay in the palm of the hand and the thumb presses on the flatter surface. In addition, for KMTBs, the position of the negative of TB is important [32]. During use (longitudinal cutting-in direction), the convex surface (top side) that possesses the negative of TB does not face the user. Therefore, the blanks of TBs as waste product of the TB performance also indicate laterality. The precise handling of hand-held KMs and KMTBs (position of fingers, thumb and hand’s palm) may also be related to natural or produced recessed grips which may reveal evidence of precise handling.

### General production sequence

The general succession of production stages for KMTBs is well understood [11, 17] and based on studies of a multitude of scholars [1, 2, 6, 7, 32, 33, 37, 44]. It combines sequential stages for all possible matrices (Table 4).

**Table 4:** Sequential steps necessary in the production sequence of *Keilmesser* with tranchet blow.

Succession	Description	Requirement
1	Selection of a suitable matrix (raw piece, core, frost shard or blank), including: - determination of the back position - determination of top and bottom side	Yes
2	Roughing-out or coarse shaping of the matrix	Only if necessary
3	Production of a back or using a natural back (cortical or old surface)	Yes
4	Shaping of the flatter surface (bottom side) or using a flat surface (e.g., ventral face)	Only if necessary
5	Shaping of the more convex surface (top side)	Only if necessary
6	Production of a striking platform at the bow (truncation) or using an existing truncation-like surface	Yes
7	Bifacial shaping of a lateral crest on the future active edge	Only if necessary
8	Production of crests or an adequate convexity on the top side for guiding the TB	Only if necessary
9	Removal of the blank of TB	Yes
10	Terminal thinning on the top side	Only if necessary
11	Regularization of the active edge which can have a bipartition (primary active edge is straight in lateral view and secondary is wavy or denticulated)	Only if necessary
12	Re-confection (reshaping and remolding)	Only if necessary

### Recognition of production sequences

For the recognition of production processes via subsequent production stages, the Working Stage Analysis (WSA, *Arbeitsschrittanalyse*, *Herstellungsanalyse*) was applied [32, 45–50]. The WSA results in clusters of related negatives (each cluster is seen as one working stage) that are indicated in the illustrations of the tools by colors. With the aid of Harris matrices the production sequence (showing the sequence of the working stages) is reconstructed [32]. Additionally, the techno-functional analysis (TFA, [38, 51]) offers evidence for classifying specific parts of the tools (such as the active edge for transformation; or bow, back and base for holding purposes). The techno-function is usually related to the working stages in that a techno-functional part is often formed by one or more working stages.

### Dimensional data collection

Three different types of distance measurement are applied to the analyzed material. 1) Direct measurement of maximum dimensions (maximum length, width and thickness) using an electronic caliper (accuracy 0.1 mm); 2) Measurement of outline parts of the KMTB (active edge, bow, back and base) after the application of WSA and TFA (accuracy 1 mm) and 3) automated measurement using freeware *Tomato Analyser* [52–54] for measuring perimeter, area and symmetry (accuracy 1 mm). Additionally, angles of the active edge and on the blanks of TBs are measured using a manual goniometer (accuracy 1°). The applied measurements are displayed in Fig 4a.

## Results of production sequence reconstruction

### Raw material

**Fig 6:**
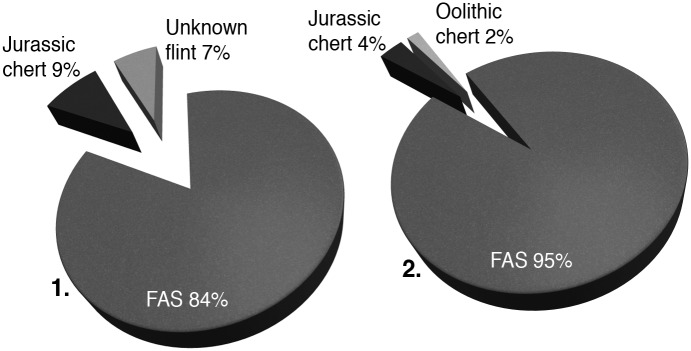
Percentage quantity of lithic raw materials used for the production for *Keilmesser* with tranchet blow (left) and blanks of tranchet blows (right). Data is listed in S1 Table.

The majority (84%, n = 36, Fig 6 and S1 Table) of the KMTBs are made from local flint (flint from the *argiles à silex*, FAS). Nowadays this material is available in close proximity on hill ranges around the site. Another n = 4 KMTBs are made from local Jurassic chert (very likely from the next valley to the north with a distance of 3 to 4 km) and the remaining n = 3 KMTBs are made from a variety of flint yet unknown. The percentage distribution of raw material for the n = 55 blanks of TBs is very similar. There are n = 52 blanks of TBs made from local FAS, n = 2 are made from local Jurassic chert and one from an oolithic chert variety of an unknown source.

### Matrix preference

**Fig 7:**
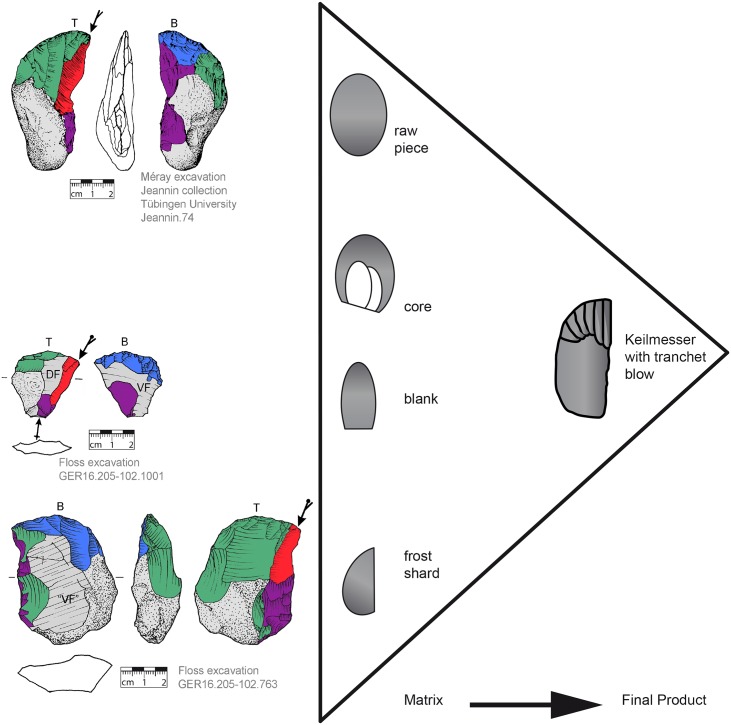
Matrices used for the production of *Keilmesser* with tranchet blow. Examples on the left (top left—cortical nodule, middle left—blanks and bottom left—frost shard) and equifinality scheme on the right.

The KMTBs show a vast range of matrices used for production (illustrated in Fig 7). Predominantly, flakes (n = 33) were used, followed by frost shards (n = 6) and one blade. Raw pieces completely covered with cortex (n = 3) are rarely used for shaping KMTBs. The preference of flakes as matrix documents their deliberate selection. It is thus likely that they were selected economically due to already existing characteristics (cortical back, asymmetric and wedge-shaped cross-section, etc.).

### Flexibility of production step succession

**Fig 8:**
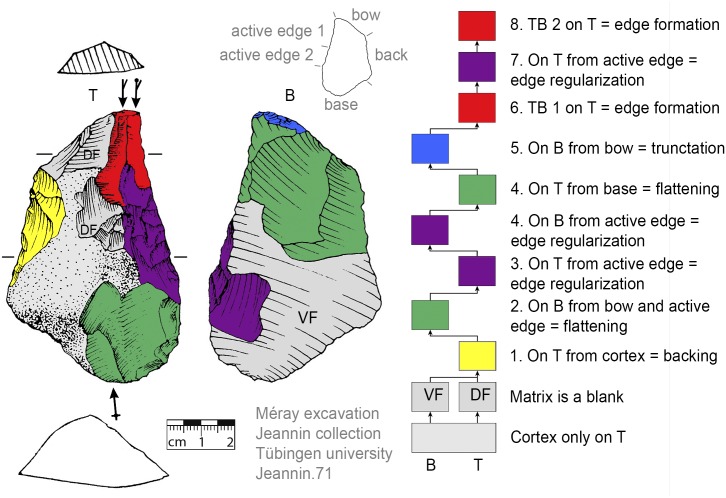
*Keilmesser* with tranchet blow with cortical back. A cortical blank is used as matrix, part of the cortical back are shaped by backing (Jeannin.71). Meaning of the colors: green = flattening (surface shaping in general, including shaping of convexities and concavities); blue = truncation (platform formation); violet = edge formation (formation of the active edge and edge regularization); yellow = backing; red = tranchet blow performance; gray = surfaces of the matrix (cortical surfaces, rest of ventral or dorsal face, etc.); B = bottom side (flatter side without tranchet blow); T = top side (convex side with tranchet blow). The Harris matrix shows the working stages succession (bottom-up). The techno-functional units of the outline of the piece are also depicted (top left). Flagged arrows on the tranchet blow signal the technique used (spherical end = direct-hard blow, circle end = direct-soft blow, without end = basal part of the negative of the tranchet blow is missing). The color and symbol schemes are also used for all other depicted *Keilmesser* with tranchet blow (Figs 9–23, 25 and 27–29).

**Fig 9:**
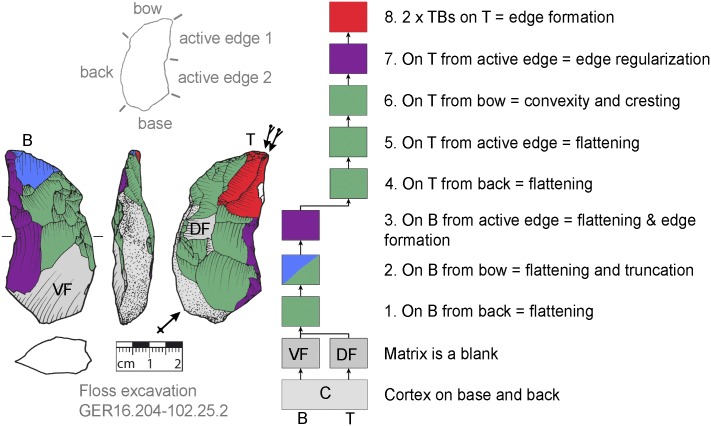
*Keilmesser* with tranchet blow with cortical back. **A** cortical blank is used as matrix, backing was not necessary (81.12.1.147).

**Fig 10:**
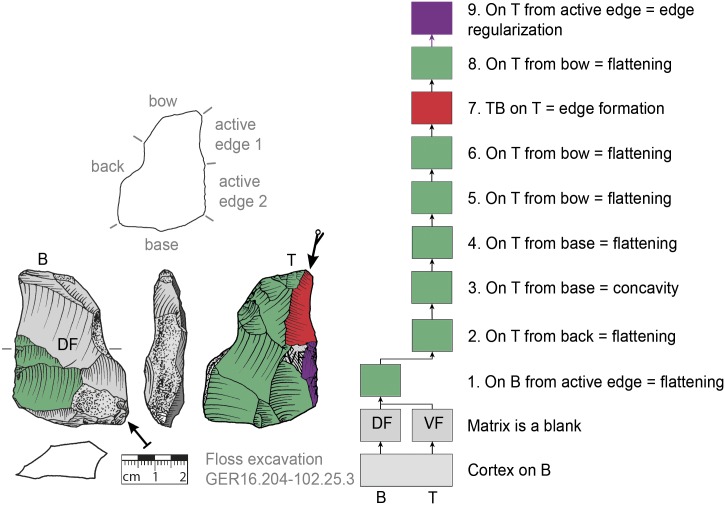
*Keilmesser* with tranchet blow with cortical back. **A** cortical blank is used as matrix, backing was not necessary (GER16.204-102.25.3).

**Fig 11:**
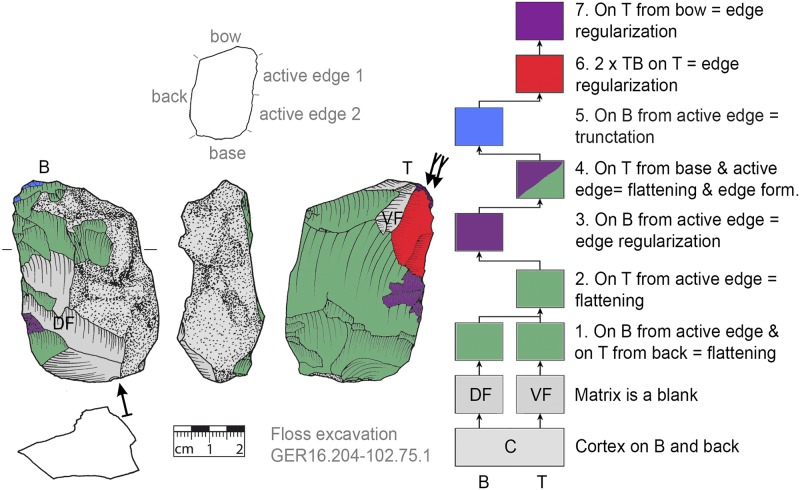
*Keilmesser* with tranchet blow with cortical back. **A** cortical blank is used as matrix, backing was not necessary (GER16.204-102.75.1).

**Fig 12:**
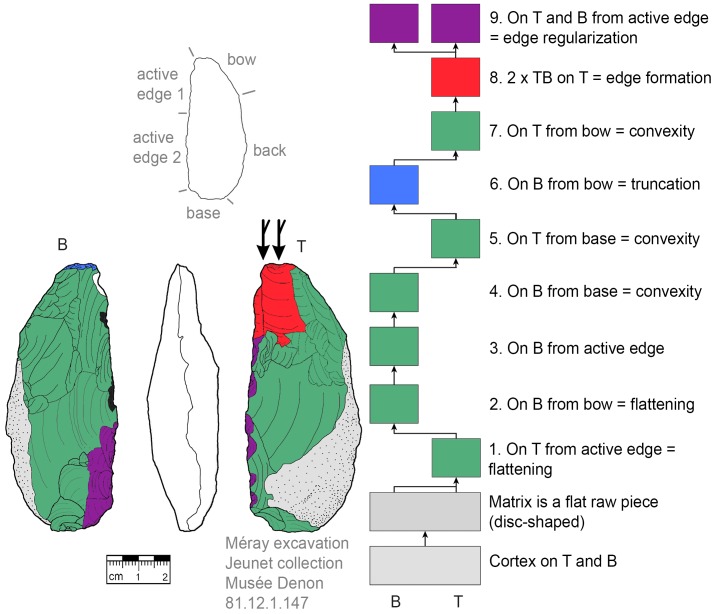
*Keilmesser* with tranchet blow with cortical back. **A** disc-shaped raw piece is used as matrix, backing was not necessary (81.12.1.147).

**Fig 13:**
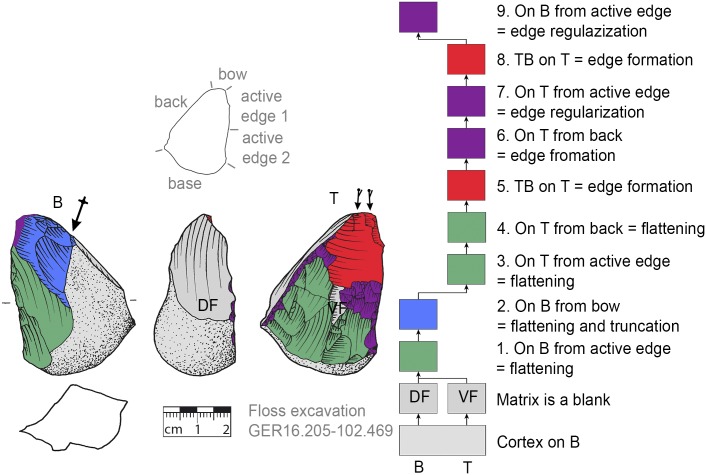
*Keilmesser* with tranchet blow with cortical back. **A** cortical blank is used as matrix, part of the back is formed by former dorsal face of the blank (GER16.205-102.469).

**Fig 14:**
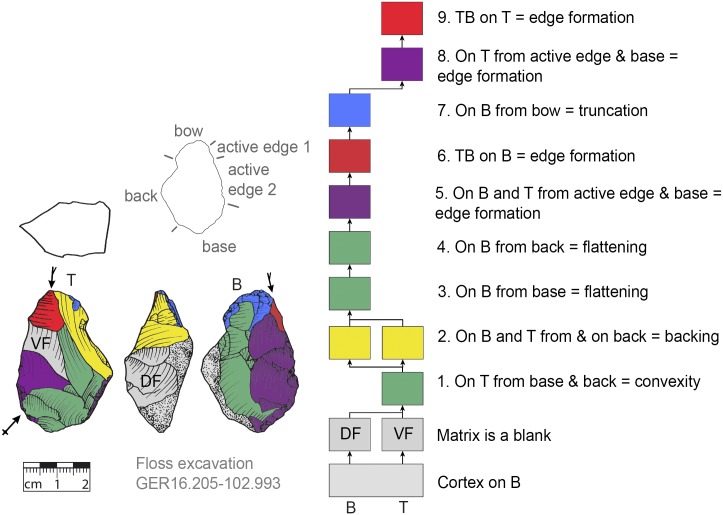
*Keilmesser* with tranchet blow with cortical back. **A** cortical blank is used as matrix, back is formed by cortex, negatives of the former dorsal face and shaped negatives (GER16.205-102.993).

**Fig 15:**
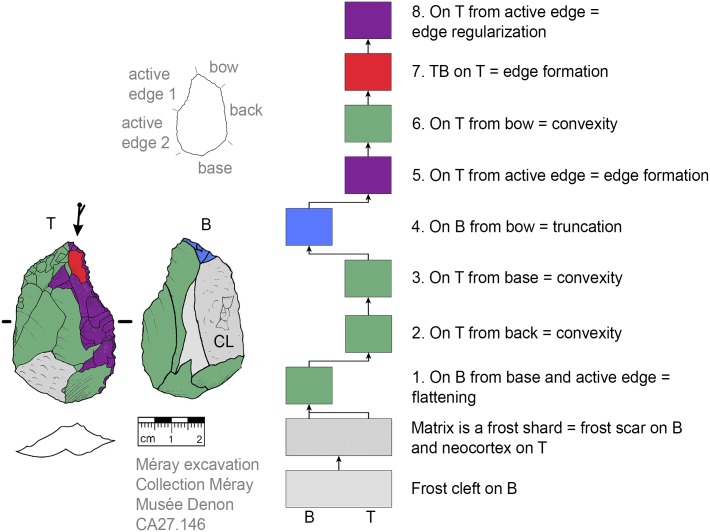
*Keilmesser* with tranchet blow using a cleft surface as back (CA27.146).

**Fig 16:**
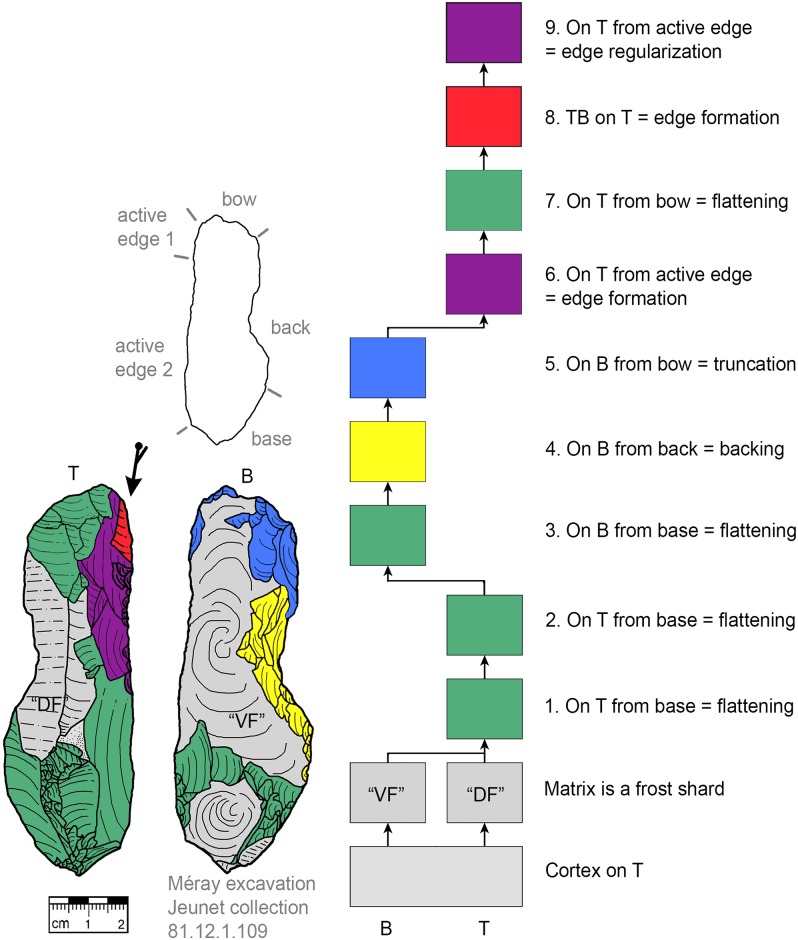
*Keilmesser* with tranchet blow with late backing (81.12.1.109). A blade-shaped frost shard is used as matrix, backing is performed late in the working stage succession.

**Fig 17:**
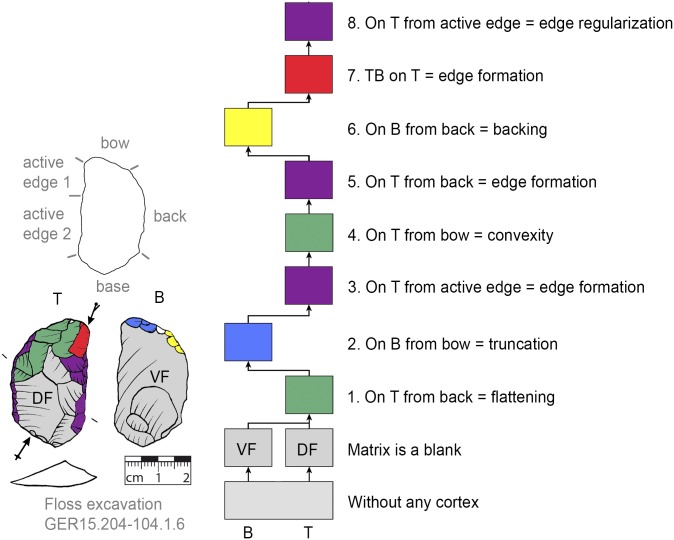
*Keilmesser* with tranchet blow with late backing (GER15.204-104.1.6). A decorticated blank is used as matrix, backing is performed late in the working stage succession.

**Fig 18:**
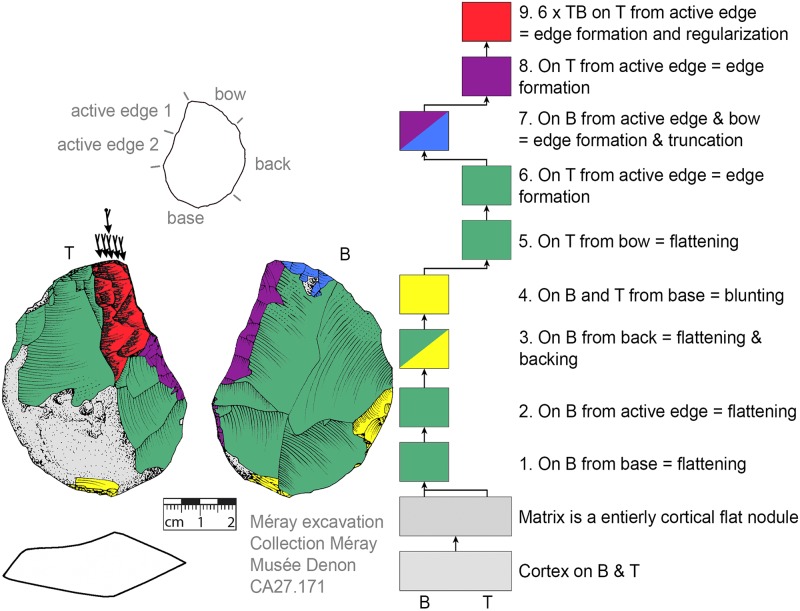
*Keilmesser* with tranchet blow with late backing (CA27.171). A flat cortical nodule was used as matrix, backing was performed after surface shaping.

The ‘standardized’ production stage sequence (see section general production sequence) suggests that a back had to be formed after the selection of suitable matrices (if none that fits was present). At VP I the majority of KMTBs possess a cortical (mostly unworked) back (n = 28, Figs 8–12). On n = 4 a surface (remaining from blank production) forms the back (Figs 13 and 14). One back is formed by a cleft surface (Fig 15). The remaining n = 10 KMTBs show a produced back. Nevertheless, (regardless of whether it is intentionally created or naturally existing), the back is the initial point of KMTB shaping on almost all objects. Only on n = 3 backing is done much later in the production sequence (Figs 16–18).

**Fig 19:**
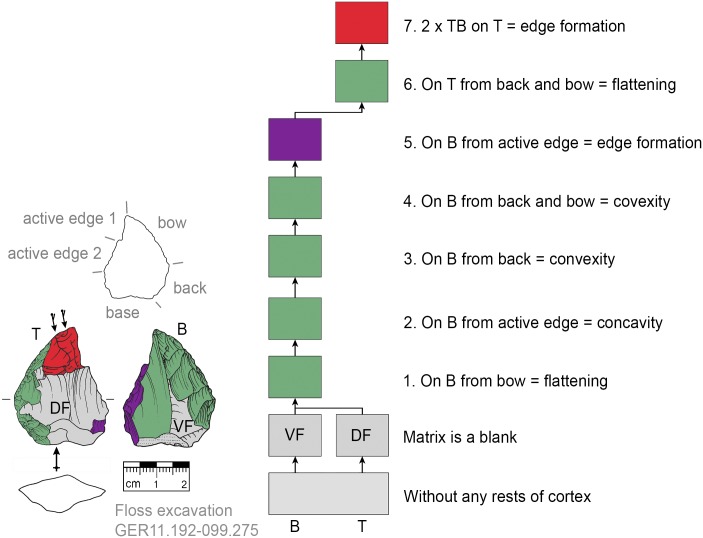
*Keilmesser* with tranchet blow showing shaping after backing. The flatter bottom side is shaped directly after bow and back formation (GER11.192-099.275).

**Fig 20:**
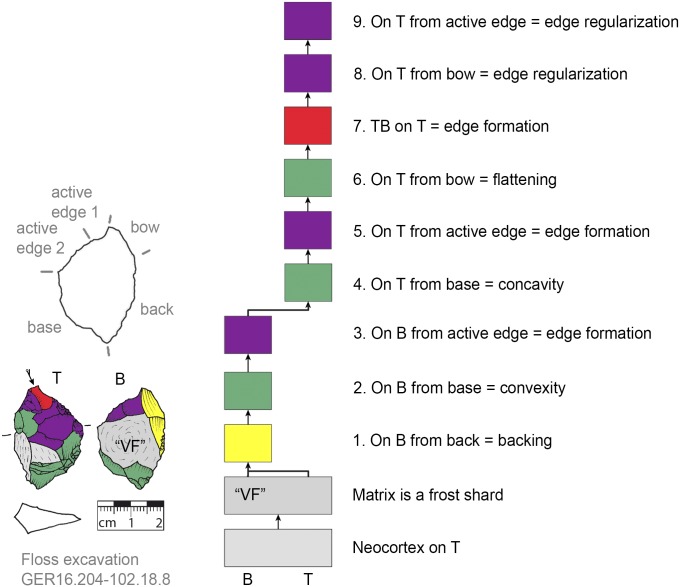
*Keilmesser* with tranchet blow showing shaping after backing. The flatter bottom side is shaped directly after backing (GER16.204-102.18.8).

**Fig 21:**
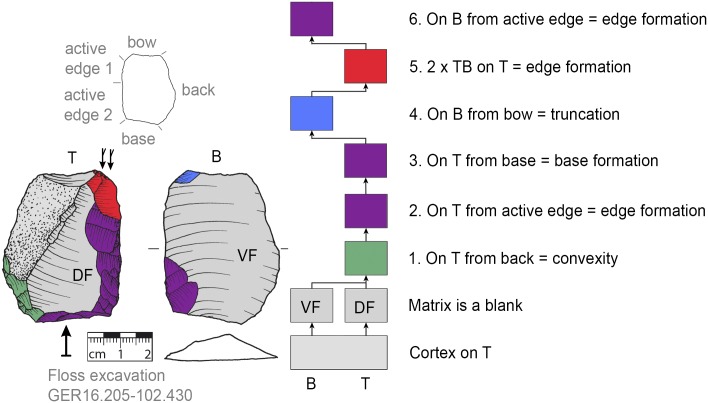
*Keilmesser* with tranchet blow showing shaping after backing. The more convex top side is shaped directly after shaping a convexity on part of the back (GER16.205-102.430).

**Fig 22:**
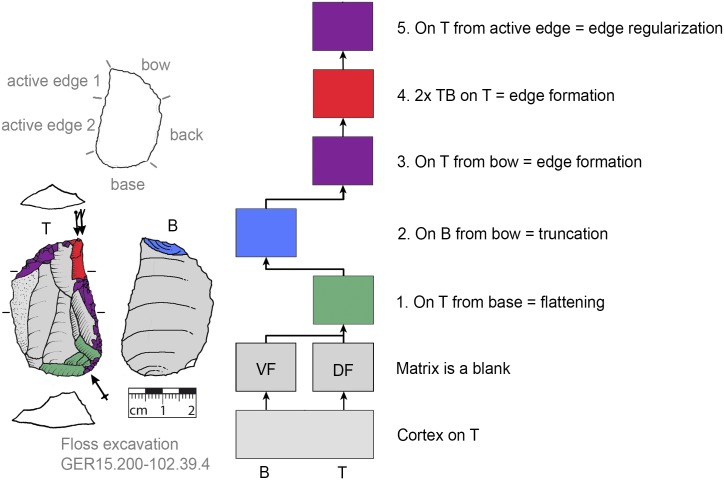
*Keilmesser* with tranchet blow showing shaping without backing. The more convex top side is shaped directly without backing (GER16.205-102.430).

**Fig 23:**
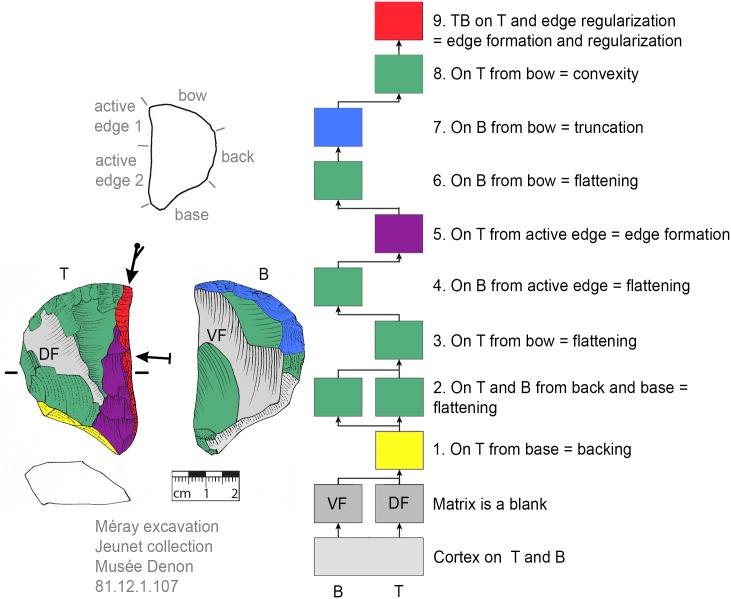
*Keilmesser* with tranchet blow showing shaping after backing. Either side is shaped directly after backing (81.12.1.107).

After the shaping of the back, surfaces are shaped. The ‘standard’ production sequence suggests the flatter side (called bottom side) should be shaped first. This procedure is visible on n = 29 KMTBs (see example in Figs 19 and 20). On another n = 14 the more convex side (top side) is shaped first (see example in Figs 21 and 22). This might provide evidence for two distinctive production branches. However, it must be taken into account that on blanks the ventral face is normally quite flat and thus does not need any flattening processes. There is one example where both sides are shaped alternately after backing (Fig 23).

The surface where the negative of TB will be situated is shaped before the TB performance. In doing so, some negatives are created on the more convex surface (i.e., top side) from the truncation, moving gradually towards the active edge, as guiding crests (schematically represented in Fig 4a). This succession of negatives is very clearly visible on a KMTB from the Jeannin collection (see Fig 7 top left).

To support the guidance of the TB (as described above) blunting of the active edge can be performed. Such a blunted edge will normally be removed by the TB, but sometimes blunted remains on the terminal end of the negative of TB are visible (examples in Figs 13 and 22). Analogous to these blunted remains on KMTBs, such an edge design is clearly visible on blanks of TBs. At VP I, there is evidence of blunting on n = 17 blanks of TBs. After performing the TB, the active edge can be regularized to remove irregularities and for straightening the cutting edge using unifacial or bifacial retouch. This regularization can be performed immediately after production or is a task for marginal corrections during use without the need of complete maintenance. More than half of the KMTBs possess such (n = 25, examples in Figs 8, 12, 13, 15–17 and 20–22).

### Lateral preference

**Fig 24:**
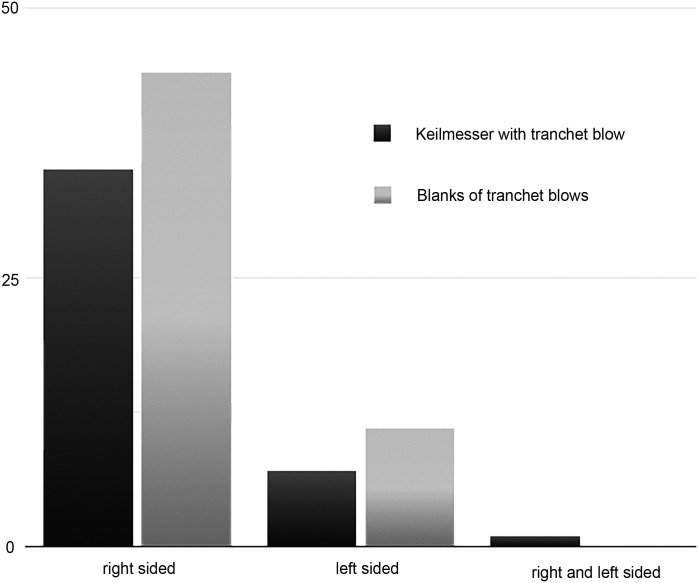
Laterality of *Keilmesser* with tranchet blow (dark gray) and blanks of tranchet blows (light gray) from Grotte de la Verpillière I (data see S2 Table).

**Fig 25:**
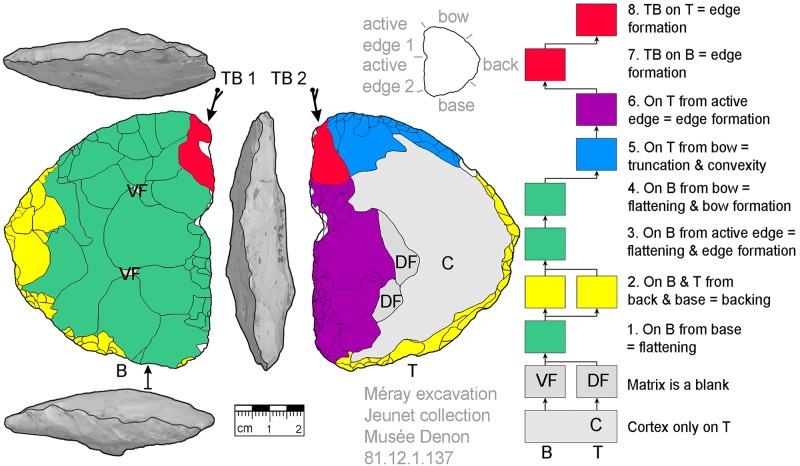
*Keilmesser* with tranchet blow from the Jeunet collection showing negatives of tranchet blows on either side on an almost entirely shaped blank (81.12.1.137).

VP I yields both left- and right-sided KMTBs, and blanks of TBs (see explanations in section Laterality and Fig 24). There are n = 7 left-sided KMTBs and n = 35 right-sided ones. The installation of TBs on either side on the same active edge is only present on one KMTB from the Jeunet collection (see Fig 25). In addition, there is evidence of n = 11 left-sided and n = 44 right-sided blanks of TBs. The majority is therefore right-sided (each around 80%, see Fig 24 and S2 Table).

### Maintenance

**Fig 26:**
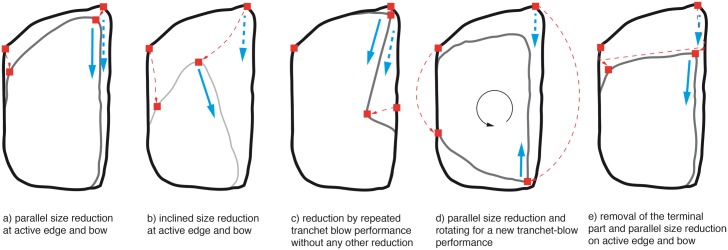
Morphology change of *Keilmesser* with tranchet blow if broken or dull, and subsequently reworked and re-sharpened, after experimentally studies of Migal & Urbanowski [44] and observations of Jöris [32]. Blue arrows indicate previous (dashed) and subsequent (solid) tranchet blows. Red squares and dashed arrows show the shift of morphological points on the outline. a) Parallel size reduction at active edge and bow, the subsequent tranchet blow is performed parallelly to the previous one; b) Inclined size reduction at active edge and bow resulting in an inclined direction of the subsequent tranchet blow; c) Reduction by repeated and parallel tranchet blow performance without any other reduction; d) Parallel size reduction and rotation for a new tranchet blow performance resulting in an opposing subsequent tranchet blow; e) Removal of the terminal part and parallel size reduction on active edge and bow resulting in a parallel subsequent tranchet blow.

Intentional drastic morphological changes after the actual production of a KMTB are related to maintenance processes after use. Five of these could be discerned (see Fig 26), whereas Migal & Urbanowski [44] experimentally studied four of them (Fig 26a–26d). In addition, Jöris [32] described the intentional removal of tool tips (break-off) and subsequent remolding of KMTBs from Buhlen, which is another maintenance process (see Fig 26e).

**Fig 27:**
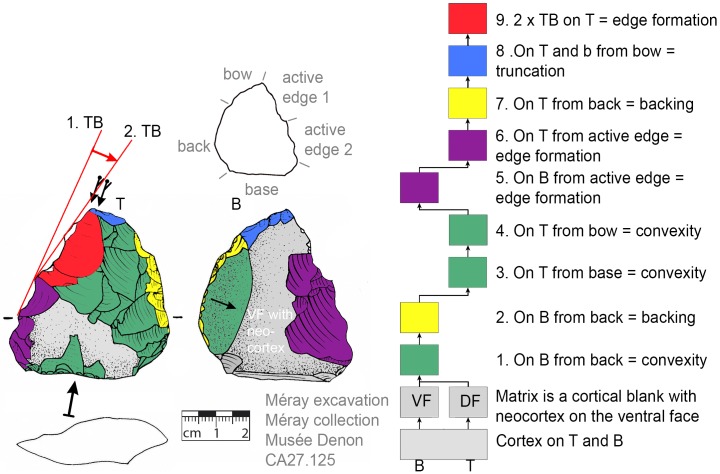
Morphology change by inclined size reduction (CA27.125). The second tranchet blow inclined the active edge (the white areas signal recent damage).

**Fig 28:**
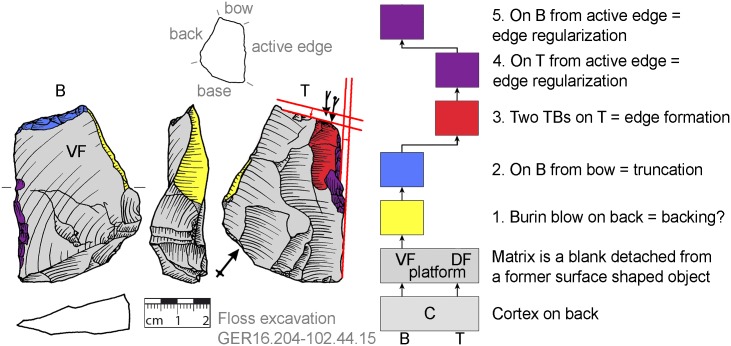
Morphology change by parallel size reduction and rotation for a new tranchet blow performance (GER16.204-102.44.15). A blank detached from a surface shaped object was used as matrix for a *Keilmesser* with tranchet blow.

**Fig 29:**
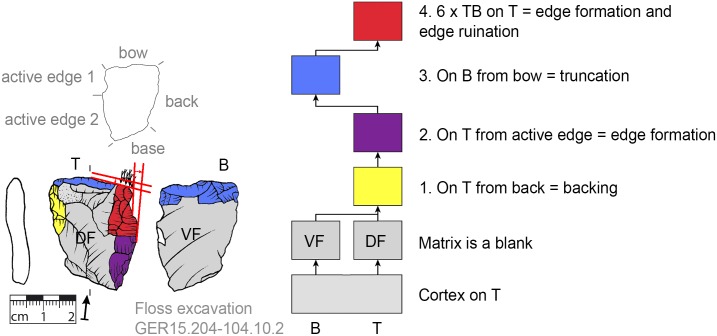
Morphology change by removal of the terminal part and parallel size reduction on active edge and bow (GER15.204-104.10.2).

First, parallel size reduction, shifting back the bow and active edge can be performed (Fig 26a and example in Fig 9). Another possibility is inclined size reduction which also takes back the bow and active edge and changes the morphology more drastically (Fig 26b and example in Fig 27). The third possibility is the immediate performance of a new TB without intensive (parallel) reduction at the bow and the active edge (for a new TB, slight reduction on the truncation might be necessary) that inclines the active edge (Fig 26c and example in Fig 7 top left). Parallel or inclined size reduction in combination with rotation is another possibility (Fig 26d and example in Fig 28). Furthermore, the removal of the terminal end of a KMTB (intentional break or caused by use) and the installation of a new bow and active edge is possible (Fig 26e and example in Fig 29).

The described maintenance processes for KMTBs can also be linked to two forming branches [11, 39]:

- remolding (remodeling, change of morphology, object becomes transformed into another tool, non-homothetic reduction process)
- reshaping (change of morphology, but tool will mostly stay the same, just smaller and slightly changed, homothetic reduction process)

Remolding or reshaping can be done immediately (reuse) or after a certain time span (recycling):

- recycling (with a hiatus in time, for instance visible via double patination)
- reuse (without a hiatus in time, immediate re-use of a device)

In that respect, the performance of a new TB to sharpen a dull active edge would be a good example for reshaping (with or without a hiatus in time). The former and latter tool is the same, it will have the same function (see Fig 8). The opposite is true for a tool that was remolded from a KMTB to a side scraper or the remolding of a bifacial object to a KMTB (see Fig 28). The performance of a TB can also create a tool that is different to the former. As *Gedankenexperiment* we can think about an abrupt retouched side scraper for transversal scraping that was modified (remolded) with a tranchet blow and used as knife for longitudinal cutting. However, it is also conceivable that a KMTB could be reworked by abrupt retouching and thus become a side scraper.

### Size comparison

**Fig 30:**
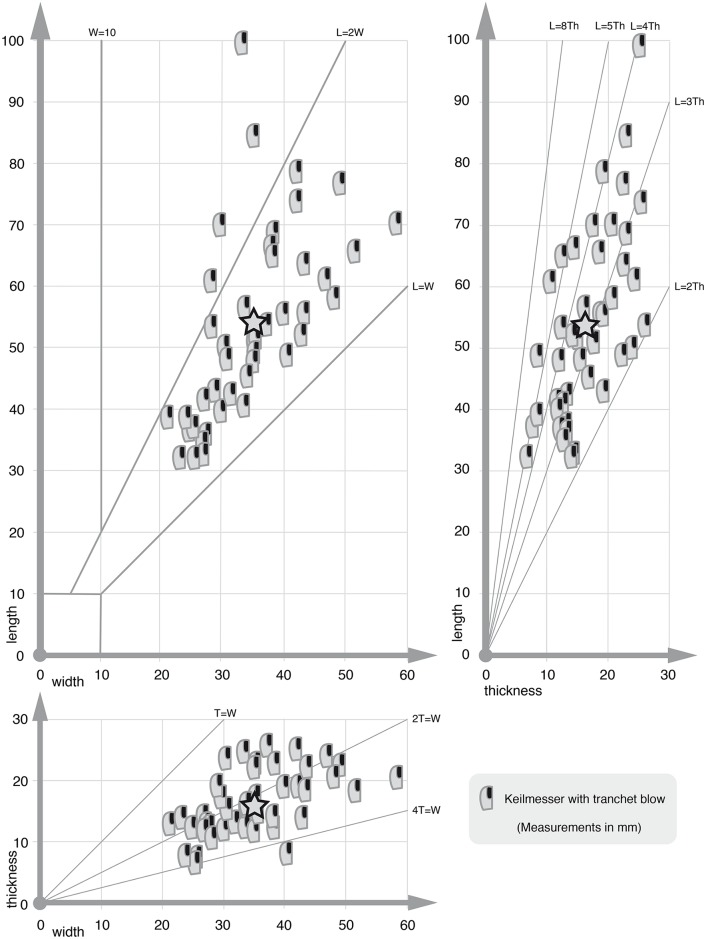
Dimension scatter plot of *Keilmesser* with tranchet blow from Grotte de la Verpillière I (length, width and thickness). The star icons mark the mean dimensions. See data in S3 Table.

**Fig 31:**
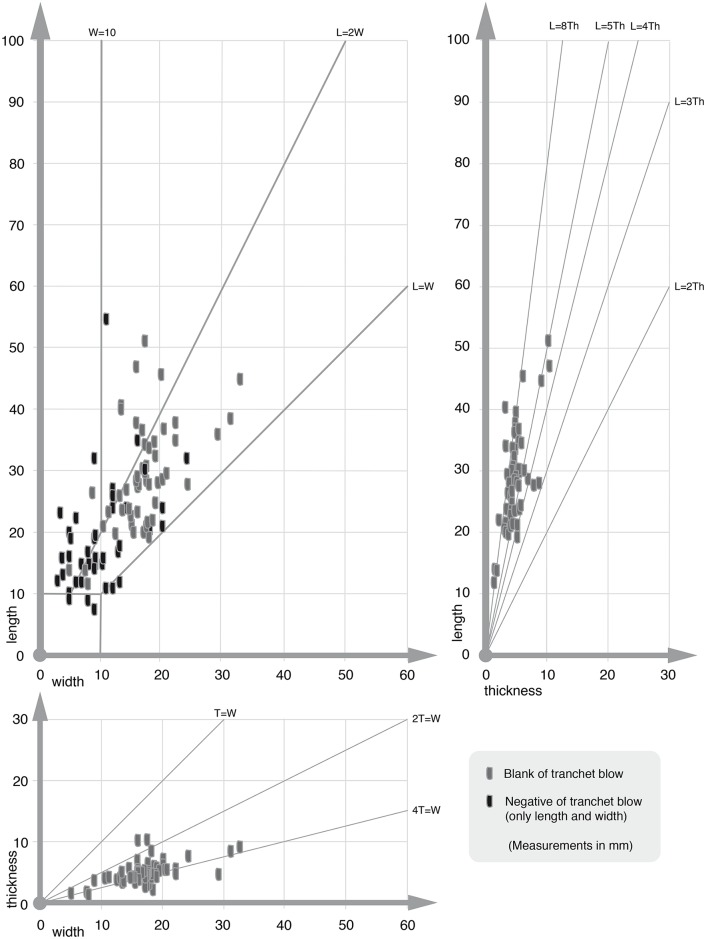
Dimension scatter plot of blanks of tranchet blows (gray) and negatives of tranchet blows (black, only length and width) from Grotte de la Verpillière I. See data in S4 and S5 Tables.

KMTBs from VP I vary in size (scatter plots in Figs 30 and 31, see also S3 and S4 Tables). The maximum-length mean is 53.7 mm, the width mean is 34.6 mm and the thickness mean is 16.3 mm (see star icon in Fig 30). The mean size of the negatives of TBs on the KMTBs is 18.96 mm times 10.09 mm (if the largest negative on each KMTB is taken into account) and if all negatives of TBs are taken into account the mean varies only slightly with 18.96 mm times 9.85 mm. The evaluation of the volume’s thickness of the negatives of TBs is too inaccurate to be taken into account.

The length of the entire active edge (measurement positions see Fig 4) varies from 22.0 mm to 88.9 mm (mean of 41.4 mm). The bow varies between 0 (zero) and 39.3 mm (mean of 22.5 mm), the back between 13.8 mm and 76.2 mm (mean of 38.2 mm) and the base between 8.7 mm and 43.3 mm (mean of 25.2 mm).

In regard to blank of TB dimensions (see Fig 18), their length ranges from 11.8 to 51.2 mm (mean of 28.6 mm). The width is situated between 4.9 and 32.5 mm (mean of 16.7 mm), and the thickness is between 1.9 and 9.0 mm (mean of 4.8 mm). A linear correlation between length and thickness is visible for most of the blanks of TBs situated at around L = 8T (see Fig 31 right above).

The dimensional range of blanks of TBs compared to the range of the negatives of TBs shows that they overlap but are not congruent to each other (Fig 31). This is technologically consistent and makes sense for the width comparison of both, because the TB performance removes material from the active edge and the resulting negative of TB on a KMTB is always narrower than the removed blank of TB. The length bandwidth of the negatives of TBs is slightly different to that of the blanks of TBs. This could be a hint that the assemblage of KMTBs and supposed corresponding blanks of TBs is not complete. This impression is supported by the fact that up to now all refitting attempts between KMTBs and blanks of TBs were unsuccessful.

**Fig 32:**
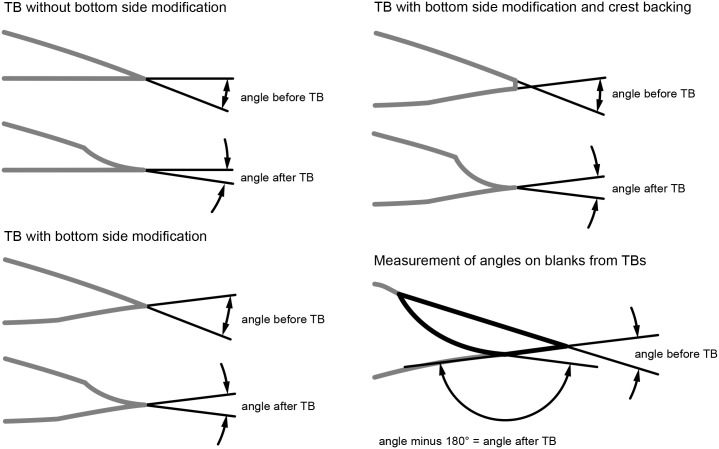
Scheme of active edge angle measurement on *Keilmesser* with tranchet blow and blanks of tranchet blows.

**Fig 33:**
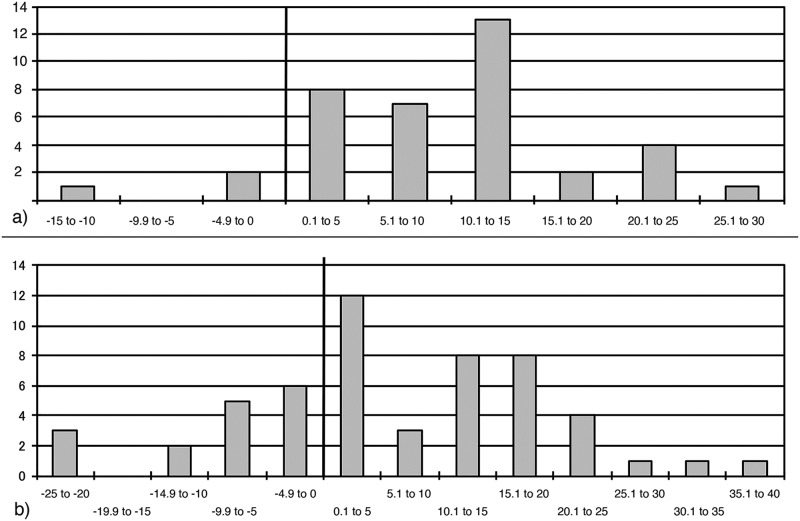
Degree differences of active edge angles before and after the tranchet blow performance on *Keilmesser* with tranchet blow (a) and blanks from tranchet blows (b) from Grotte de la Verpillière I.

The angle magnitude before and after the TB performance on the active edge was measured on n = 43 KMTBs (see scheme in Fig 32). It varies between 46° and 79° (mean of 64.97°) before the TB and between 35° and 85° (mean of 55.8°) after it. On three KMTBs the edge-angle became larger (Fig 33a, minus values on the left). A peak of angle difference is situated between 10.1° and 15° (see Fig 33a, plus values on the right).

The measurement of the edge angle on blanks of TBs is somewhat different to the performance on KMTBs (see scheme right below in Fig 32). It varies on the blanks of TBs before the TB between 28° and 89° (mean of 52,96°) and after the TB between 24° and 38° (mean of 58,46°). On n = 16 blanks of TBs the edge-angle became greater (Fig 33b, minus values on the left). A peak of angle difference is situated between 0.1° and 5° (see Fig 33b, plus values on the right).

All KMTBs together possess n = 77 negatives of TBs, including n = 20 KMTBs with one negative of TB, n = 20 KMTBs with two negatives of TBs, one KMTB with n = 5 negatives of TBs and two KMTBs with n = 6 subsequent negatives of TBs.

### Outline analysis

**Fig 34:**
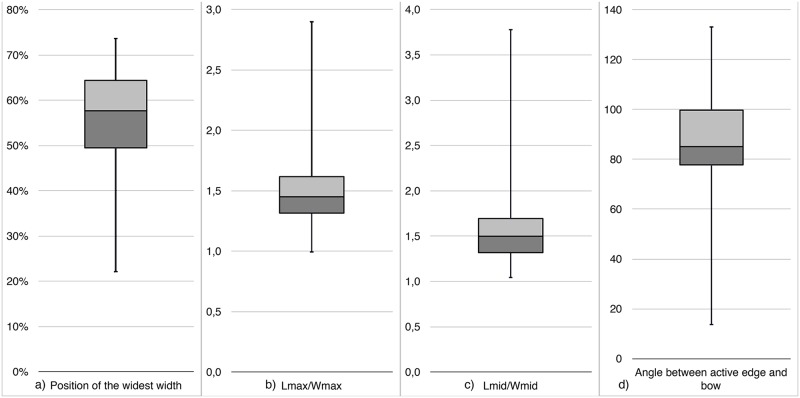
Selected results of the outline and area analysis of the *Keilmesser* with tranchet blow with the aid of *Tomato Analyser*, displayed as box plots. a) Position of the widest width in percentage of the maximum length; b) Relation between maximum length and maximum width; c) Relation between mid-length and mid-width and d) Angle between active edge and bow.

The outline and area analysis created with the aid of the free software *Tomato Analyser* revealed the following relations: The comparison of the area above and below mid width of the KMTBs showed that n = 32 have a greater area below and n = 11 above the mid width. This is in opposition to the position of the widest width on the KMTBs. There, nearly 75% possess their widest width above 1/2 of the maximum length (see Fig 34a). The relation between the maximum length and maximum width is displayed in Fig 34b and shows that the average is at 1.45, whereas the average of medium length to medium width is 1.5 (Fig 34c). The average angle between the active edge and the bow on the KMTBs is 85.1°, but ranges from 13.6 to 133.1° (see Fig 34d).

## Discussion

Despite the obvious morphological differences within the KMTBs, there is a high congruence in the presence of technological features visible. Such technological features are essential details necessary to perform a TB (striking platform and convexity that can be removed during the TB performance). Some of these differences can be explained by the matrix used, as well as maintenance processes.

### Matrix

One reason for the diversity of bifacial objects that were modified with a TB is clearly associated with different matrices (fr. *support*, [55]) used as a basis for production and the different needs of shaping for getting the wedge shape in cross section and a straight cutting edge (Fig 4). In addition to lithic raw pieces (nodules), frost shards and blanks can be selected to realize the concept KMTB (see also Fig 7).

A nodule seldom possesses a plano-convex cross section, thus making it necessary, at least on the terminal part, to create one. As visible in Fig 7 (top left), back and base are completely unworked. Only the terminal part is highly worked. The opposite is visible on Fig 12. Here, a nodule is also used, but the surface on the bottom side is completely worked.

On blanks, despite the plano-convex cross section, the bottom side (very often the former ventral face) is often entirely reworked.

The predominant use of blanks (i.e. flakes and blades) as matrix is also known from other sites. For instance, Bourguignon [13] reports this from Abri du Musée (Les Eyzies, France) and also Krukowski [1] described it for material from Ciemna (Poland). In contrast, at Buhlen flat raw pieces are preferred [32]. If blanks are available for the production, the benefit is that blanks with a very specific morphology can be selected, possessing an asymmetrical but plano-convex cross-section, because such a morphology is already very close to the resulting shape of a KMTB. Thus, the performance of some production steps is not necessary and differs from the shaping of raw pieces.

### Interchangeability of working stages

**Fig 35:**
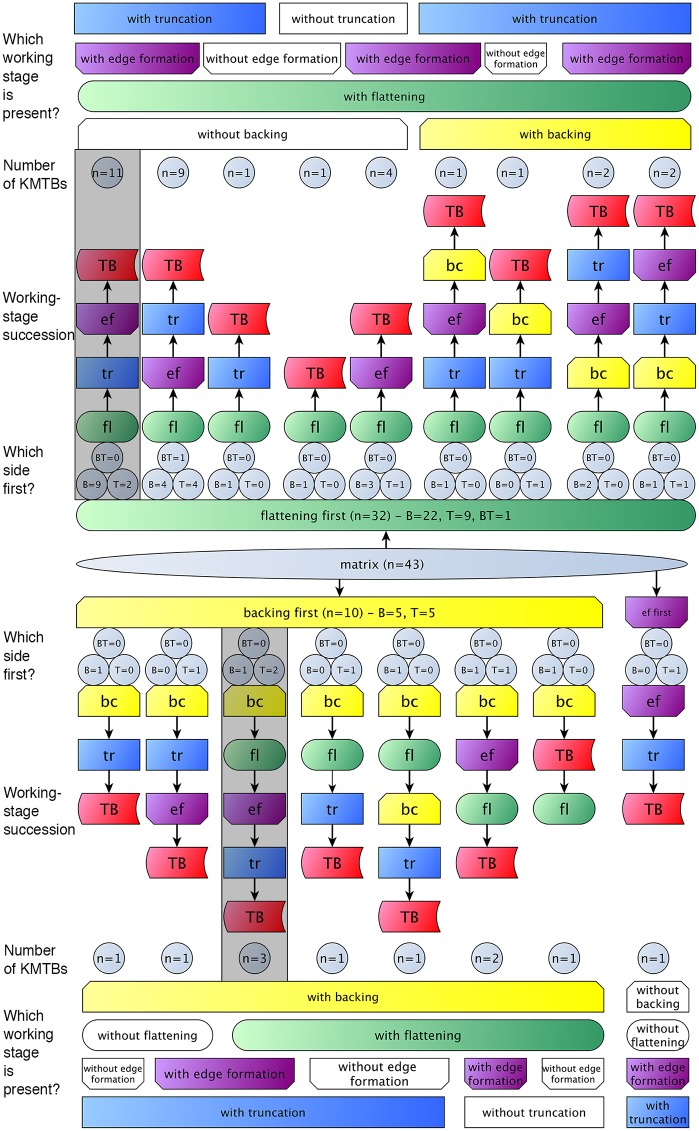
Comparison of working stage succession on all *Keilmesser* with tranchet blow from Grotte de la Verpillière I. Gray shaded field on top indicates the working stage succession which is prevalent at VP I and the gray shaded field below marks the main working stage succession at the Buhlen site (abbreviations: fl = flattening (surface shaping in general, green); tr = truncation (platform formation, blue); ef = edge formation (prevalently the formation of the active edge, violet); bc = backing (yellow); TB = performance of a tranchet blow (red); B = bottom side shaped first; T = top side shaped first and BT = indistinguishable if bottom or top side is shaped first).

A comparison of the sequences of individual working stages and their assumed reasons reveals the high variability of the KMTB concept (Fig 35). There are two main groups for the first step executed (regardless of the matrix used).

The larger group contains objects where the flattening (or more general surface shaping) is done first, regardless of whether a back is created later or a natural back is present (see top half of Fig 35).

The other group contains pieces where the back is formed first (see bottom half on Fig 35). In both groups, however, the following steps are interchangeable, assuming that a truncation is available as a striking platform in order to be able to perform the TB (usually a created truncation). The comparison of the sequence of the working stages also shows that the ‘classic’ production sequence, as shown by Jöris [32] for the material from Buhlen, is rarely found at VP I (gray shaded succession at the bottom half in Fig 35). The larger group of objects first shows flattening, followed by truncation, edge formation and TB performance (gray shaded succession at the top half in Fig 35).

In the course of discussing exchangeability of working stages we are confronted with the problem of equifinality [56]. Equifinality can be described as follows: “*Viewed as a system*, *the fracture of flintlike materials exhibits the property of equifinality—a characteristic of open systems*. *In other words*, *the same final state may be reached from different initial conditions and in different ways* […]” [57].

In this context, the performance of a tranchet blow is the final step.

There are special morphologies of surfaces and edges on lithic objects that are necessary to be able to perform a TB, regardless of the matrix used. The decisive criterion is the presence of these morphologies, irrespective of whether they are already in existence or whether they are created during the production process. The most important parameter is the condition of the edge between the truncation and the top side, and therefore, the constellation of how these surfaces meet each other. Another parameter is the shape of the top side where the TB will remove material.

As Van Peer [58] showed for Levallois reduction, it is not important whether a convexity is produced or whether there are so-called guiding ridges. The importance lies in a general convexity to be able to extract a specific volume at the desired position. This volume and the resulting convexity is produced primarily by removing blanks from the top side along the bow in the direction of the cutting edge, as it was described by Jöris [32] or Urbanowski [33]. Thus, we conclude that, despite remarkable differences in morphology and the exchangeability of working stages, one line is being followed: the performance of a TB in order to produce a sharp straight cutting edge. Regarding the existing equifinality, we assume that the entire production follows a concept that appears with different manifestations.

### Comparison between Buhlen and Grotte de la Verpillière I

Within the available technological studies, the upper site of Buhlen provides data adequate for comparative analysis of KMTBs. As Jöris [32] published data about laterality, as well as dimensions of bow, back-base and active edge, it allows the formulation of comparisons. On the one hand, the number of left-sided objects from VP I (see Fig 14) is much higher than at the Buhlen site. There, more than 90% of the KM are right-sided and 85,4% of the blanks of TBs, respectively. On the other hand, Buhlen also yielded n = 2 “*Pradnik-Schaber*” that possess negatives of TBs on either side, a feature that is present at VP I on one KMTB (see Fig 25).

**Fig 36:**
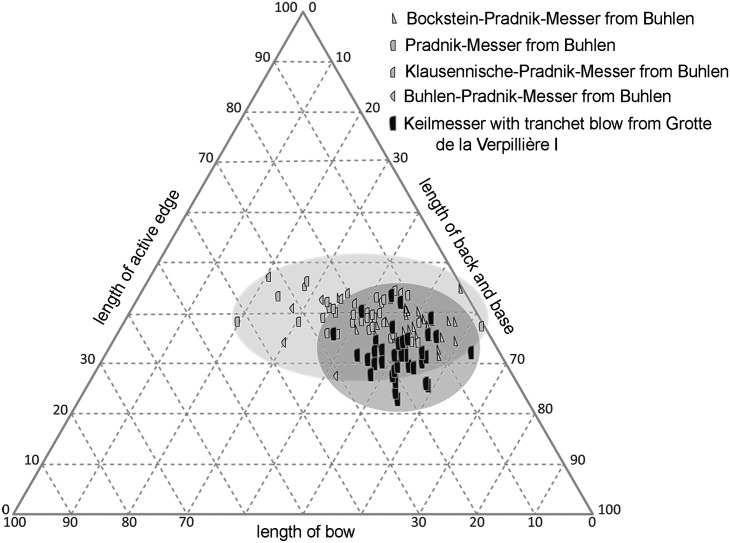
Comparison of length relations between pieces from Grotte de la Verpillière I (data see S6 Table) and Buhlen (data from Jöris [32]), displayed as triplot.

If (regardless of size) relations of parts of the outline between both sites are compared (see Fig 36), it is recognizable that the KMTBs from VP I are more clustered. At VP I, the bow is quite short in relation to the active edge and the combination of back and base (around 35 to 65%) and tendentiously at Buhlen longer (around 35 to 80%). If the active edge is taken into account, the span on both sites is quite similar (VP I—around 20 to 45%; Buhlen—around 25 to 50%).

The comparison of total length and width of the KMTBs between both sites show that the VP I pieces are nearly in the same size range as the Buhlen material. The only difference is that longer pieces at VP I are tendentiously wider than at Buhlen (see S1 Fig).

## Conclusions

The KMTB concept, as recognized at VP I, is both, highly flexible and static. The concept is flexible in the sense that different production steps are interchangeable or could even be omitted if the respective edges and surface characteristics were already present through the matrix used. The static properties of the concept are related to physical constants. For example, it is necessary that both, surfaces and edges enable the application of a TB at the intended position. Therefore, certain working stages are necessary to fashion these surfaces and edges in an appropriate shape. It is also advantageous to include adequate surfaces and edges in the working process, which can originate from previous working stages or already exist through the matrix. Consequentially, the matrices can be selected according to their specific characteristics. Among other things, the following characteristics are mandatory for the application of a TB: 1. A convexity along the future cutting edge, but only on one of the two faces; 2. The striking platform must be at an exact angle to this convexity, so that the convexity can be reduced in such a way that the future cutting edge is not blunted but its cutting angle is reduced; 3. The convexity of the surface should not be interrupted, as otherwise the blank will produce a hinge at this point, as is also the case with a burin reduction. Similar to other concepts such as Levallois, exact planning in advance is necessary, starting with the selection of the matrix.

In order to achieve the characteristics described above for applying a TB, various other edges and surfaces must also be brought into a particular shape. This concerns the bow and the future cutting edge above all. The bow is almost inevitably created by the fact that the successively formed, elongated convexity of the top side reaches the striking platform of the bottom side at the terminal end.

In order to apply a negative of tranchet blow, it is not important whether the cutting edge has been processed uni- or bifacilly, which is evident in some pieces. However, it is advantageous if the cutting edge has an asymmetrical cross-section. The shape alone, as the two surfaces meet at the cutting edge, already provides a certain convexity that can be exploited.

The actual size of the pieces only plays a minor role. Due to the selected form of the matrix, only few steps are necessary to achieve the goal of placing a negative of tranchet blow on some pieces. The actual sequence of the working stages is largely irrelevant. However, it is important not to lose sight of the goal every time a working stage is carried out. This goal-directedness can be called “faith to conception”.

For this reason, the concept implies that the final product can be achieved by means of different production paths, whereby the sequence of working stages can be interchanged. In some cases, working stages can be omitted, since the matrix already has a corresponding shape of the respective position. In many cases the practicability is also relevant, as it can be advantageous to carry out one step before another.

As can be seen in most of the pieces, the detachment of a negative of tranchet blow was not intended as an option, but as a definitive goal of production. The entire production was planned and carried out accordingly in order to achieve this goal. The idea behind the application of this negative of tranchet blow was to get a straight, sharp, low-angle cutting edge.

The KMTB concept gives the producer plenty of freedom. However, the specific physical properties must be fully understood in order to be able to produce convexities and edges in a very specific way. Of course, this can be said about the vast majority of lithic concepts. In this particular case, however, even the slightest deviation in the constellation and design of the surfaces and edges that are necessary to produce the negative of tranchet blow will immediately lead to failure. The idea behind the concept can be summed up as follows: The high flexibility of ways to create a KMTB shows a certain diversity in homogeneity (equifinality, different matrices and different production paths result in the same product), likewise integrated is a homogeneity in diversity (the same morphologies are necessary to produce a negative of tranchet blow).

As it turned out, the inventory of KMTBs and corresponding blanks of tranchet blows at VP I cannot be complete, but it proves that the negatives of tranchet blows were produced on site. The number of more than three dozen KMTBs shows that the production (including resharpening) of the pieces at this site was of some importance for the Middle Paleolithic people. The failure of refitting to date is evidence of the possible presence of further pieces at the site.

Since studies of Desbrosse [8, 24] in the 1970s, the assemblage of KMTBs and blanks of tranchet blows were massively extended (from 27 to 99 pieces). However, Desbrosse [8, 24] examined only n = 9 of the n = 27 pieces that had previously been recovered. Thus, the study carried out here underlines the relevance of the finds and shows that these are not singular elements, but perhaps even an important part of the Middle Paleolithic of the region.

In addition to the pieces presented in this study, our work now provides numerous indications for the presence of such special pieces in other localities of the Côte chalonnaise [11, 17, 59]. The presence of these surrounding sites makes it necessary to reflect on regional patterns of the late Middle Paleolithic of the region. The congruence of the sites is demonstrated by the following elements: Presence of the KMTBs phenomenon, coupled with numerous morphologically diverse bifacial objects, prevailing production of blanks using the Levallois concept, small numbers of blades and *Groszaki*, ventral reduction for the configuration of Levallois cores and bulb reduction of blanks, minor presence of other blank-production concepts, small quantities of ‘Upper Paleolithic’ tools, high diversity of modifications on cores and blanks for tool production and various evidence of hafting.

Despite potential regional clustering of the KMTB concept, its general distribution within a Late Middle Paleolithic time frame is quite limited and does to date not exceed the above mentioned 14 sites (Table 1). Therefore, the recognition and further analysis of this tool production concept with its demonstrated flexibility within static constraints, provides further insight into the technological behavior of (central) European Late Middle Paleolithic Neanderthals.

## Supporting information

S1 Table Lithic raw material of *Keilmesser* with tranchet blow and blanks of tranchet blows from Grotte de la Verpillière I. (PDF)

S2 Table Laterality of *Keilmesser* with tranchet blow and blanks of tranchet blows from Grotte de la Verpillière I. (PDF)

S3 Table Total size of *Keilmesser* with tranchet blow from Grotte de la Verpillière I. (PDF)

S4 Table Total size of blanks of tranchet blows from Grotte de la Verpillière I. (PDF)

S5 Table Size of negatives of tranchet blows on *Keilmesser* with tranchet blow from Grotte de la Verpillière I. (PDF)

S6 Table Length of active edge, bow, back and base of *Keilmesser* with tranchet blow from Grotte de la Verpillière I. (PDF)

S1 Fig Length and width comparison of *Keilmesser* with tranchet blow from Grotte de la Verpillière I with *Keilmesser* from the Buhlen site. Data of the Buhlen site from Jöris (2001).
(PDF)
