# Supplementary material for: The performance of tranchet blows at the Late Middle Paleolithic site of Grotte de la Verpillière I (Saône-et-Loire, France)
Source: PLoS One. 2017 Nov 30;12(11):e0188990. doi: 10.1371/journal.pone.0188990 (PMC5708829; doi:10.1371/journal.pone.0188990)
Supplement: S1 Table — (PDF) [file pone.0188990.s001.pdf]

S1 Table. Lithic raw material of Keilmesser with tranchet blow and blanks of tranchet blows from Grotte de la Verpillière I.

Raw material	Keilmesser with tranchet blow	Blanks from tranchet blows
**FAS** [a]	37	52
**Jurassic chert**	4	2
**Unknown flint**	3	0
**Oolithic chert**	0	1
**Total**	**44**	**55**

[a]FAS, Flint of the argiles à Silex (clay-with-Flints)
