# Supplementary material for: The performance of tranchet blows at the Late Middle Paleolithic site of Grotte de la Verpillière I (Saône-et-Loire, France)
Source: PLoS One. 2017 Nov 30;12(11):e0188990. doi: 10.1371/journal.pone.0188990 (PMC5708829; doi:10.1371/journal.pone.0188990)
Supplement: S2 Table — (PDF) [file pone.0188990.s002.pdf]

S2 Table. Laterality of Keilmesser with tranchet blow and blanks of tranchet blows from Grotte de la Verpillière I.

Laterality	Keilmesser with tranchet blow	Blanks of tranchet blows
**Left-sided**	7	11
**Left- and right-sided**	1	0
**Right-sided**	35	44
**Total**	**43**	**55**
