# Supplementary material for: The performance of tranchet blows at the Late Middle Paleolithic site of Grotte de la Verpillière I (Saône-et-Loire, France)
Source: PLoS One. 2017 Nov 30;12(11):e0188990. doi: 10.1371/journal.pone.0188990 (PMC5708829; doi:10.1371/journal.pone.0188990)
Supplement: S3 Table — (PDF) [file pone.0188990.s003.pdf]

S3 Table. Total size of Keilmesser with tranchet blow from Grotte de la Verpillière I

Inventory number (ancient finds)	Square meter (Floss excavation)	Find number (ID)	Sub find number (Suffix)	Total length	Total width	Total thickness
Jeaninn.	-	62	0	39.65	29.78	11.89
Jeaninn.	-	71	0	76.88	48.73	22.28
Jeaninn.	-	72	0	70.06	29.48	17.38
Jeaninn.	-	73	0	56.69	33.45	16.04
Jeaninn.	-	74	0	78.7	41.86	18.94
Jeaninn.	-	75	0	63.66	43.3	22.32
Jeaninn.	-	76	0	68.75	38.08	22.74
Jeaninn.	-	77	0	55.58	39.64	19.1
Jeaninn.	-	92	0	40.63	33.27	12.37
81.21.1.	-	107	0	51.7	35.38	14.88
81.21.1.	-	109	0	99.64	32.93	24.75
81.21.1.	-	135	0	60.9	27.8	10.4
81.21.1.	-	137	0	70.2	58.1	20.4
81.21.1.	-	147	0	84.6	35	22.8
CA 27	-	125	0	52.14	42.6	13.94
CA 27	-	126	0	73.78	41.78	25.22
CA 27	-	146	0	51.1	35	17.5
CA 27	-	171	0	65.72	51.29	18.44
-	192-099	275	0	35.88	27.2	13.03
-	200-102	34	6	48.2	30.4	15.3
-	200-102	39	4	41.5	26.9	11.5
-	201-105	1	19	48.0	34.8	11.8
-	204-103	4	1	34.9	26.4	12.6
-	204-104	1	4	37.6	21.0	12.9
-	204-104	1	6	38.9	23.6	8.2
-	204-104	10	2	37.2	25.3	7.5
-	214-112	1	14	65.1	38	12.3
-	214-112	2	3	66.4	37.8	14.2
-	204-102	18	8	36.8	24.5	12.3
-	204-102	23	3	32.88	26.6	14.22
-	204-102	25	2	53.5	27.7	12.2
-	204-102	25	3	53.8	36.9	25.8
-	204-102	44	15	55.7	43	18.5
-	204-102	48	4	42.5	31.5	13.2
-	204-102	75	1	61.3	46.5	24.1
-	205-102	216	0	45.3	33.5	16.6
-	205-102	330	0	50.1	30	23.6
-	205-102	430	0	48.9	40.2	8.3
-	205-102	469	0	48.9	34.9	22.1
-	205-102	539	0	-	-	-
-	205-102	615	2	32.43	22.93	13.95
-	205-102	763	0	58.3	48.1	20.5
-	205-102	993	0	43.1	28.7	19.1
-	205-102	1001	0	32.29	24.89	6.69
