# Supplementary material for: The performance of tranchet blows at the Late Middle Paleolithic site of Grotte de la Verpillière I (Saône-et-Loire, France)
Source: PLoS One. 2017 Nov 30;12(11):e0188990. doi: 10.1371/journal.pone.0188990 (PMC5708829; doi:10.1371/journal.pone.0188990)
Supplement: S4 Table — (PDF) [file pone.0188990.s004.pdf]

S4 Table. Total size of blanks of tranchet blows from Grotte de la Verpillière I.

Inventory number (ancient finds)	Square meter (Floss excavation)	Find number (ID)	Sub find number (Suffix)	Total length	Total width	Total thickness
Jeannin.	-	44	0	40.5	13.39	3.21
02.14.	-	99	0	34.58	18.53	5.9
02.14.	-	26	0	33.8	18.03	4.26
02.14.	-	142	0	34.1	17.36	3.17
Pelatin.	-	167	0	32.3	18.76	4.49
Pelatin.	-	219	0	-	-	-
Pelatin.	-	168	0	28.4	17.47	4.68
Pelatin.	-	169	0	21.97	18.36	2.17
89.78.1.	-	103	0	29.41	16.07	3.6
-	200-102	34	5	47.1	15.9	10.1
-	200-102	34	61	24.7	18.9	5.7
-	201-105	1	2	36.6	16.5	4.8
-	202-103	10	1	39.8	13.3	4.9
-	203-102	19	0	26.4	8.7	3.6
-	203-102	8	0	27.7	15.9	5.3
-	203-103	33	0	13.9	7.5	1.7
-	203-103	59	0	38.3	31.1	8.4
-	203-103	263	0	23.4	11.2	4.2
-	204-103	139	0	13.9	4.9	1.5
-	214-112	1	5	28.8	20.0	7.0
-	203-104	265	1	30.7	17.4	4.4
-	203-104	359	0	21.07	10.50	4.02
-	203-104	255	12	21.5	17.7	3.2
-	203-104	612	0	11.8	7.8	1.3
-	203-104	249	1	23.6	15.9	4.8
-	203-104	656	31	19.9	15.3	3.3
-	203-104	307	0	30.1	17.5	6.3
-	203-104	25	0	45.6	20	6
-	203-104	279	0	23.8	13.6	3.5
-	203-104	655	3	37.8	22.1	4.8
-	204-102	26	1	44.8	32.5	9.0
-	204-102	25	10	33.6	18	4.7
-	204-102	21	9	28	16	3.7
-	204-102	21	1	51.2	17.3	10.2
-	204-102	18	4	20.4	17.1	3.1
-	204-102	18	1	27.7	24.1	7.7
-	204-102	40	4	19.3	17.7	5.1
-	204-102	78	1	37.6	15.8	6.9
-	204-102	14	1	24.0	14.4	5.5
-	204-102	13	2	25.9	13.0	4.0

-	204-102	8	2	36	29	4.6
-	204-102	49	28	26.8	14.5	4.2
-	204-102	41	2	30.4	16.9	5.3
-	204-102	9	1	36.8	20.3	5.3
-	204-104	170	4	19.8	12.4	3.7
-	205-102	70	1	34.9	22.1	5.5
-	205-102	1050	4	30.2	17.1	4.3
-	205-102	980	1	28.0	18.0	8.5
-	205-102	962	8	29.8	20.6	5.5
-	205-102	427	0	24.8	18.8	4.3
-	205-102	384	0	29.0	15.8	5.0
-	205-102	456	0	21.4	14.8	4.3
-	205-102	222	0	28.1	19.4	5.0
-	205-102	7	3	22.8	15.1	4.1
-	205-102	1050	3	21.2	17.5	5.0
