# Supplementary material for: The performance of tranchet blows at the Late Middle Paleolithic site of Grotte de la Verpillière I (Saône-et-Loire, France)
Source: PLoS One. 2017 Nov 30;12(11):e0188990. doi: 10.1371/journal.pone.0188990 (PMC5708829; doi:10.1371/journal.pone.0188990)
Supplement: S5 Table — (PDF) [file pone.0188990.s005.pdf]

S5 Table. Size of negatives from tranchet blows on Keilmesser with tranchet blow from Grotte de la Verpillière I.

Inventory number (ancient finds)	Square meter (Floss excavation)	Find number (ID)	Sub find number (Suffix)	Length of the first tranchet blow negative	Width of the first tranchet blow negative	Length of the second tranchet blow negative	Width of the second tranchet blow negative	Length of the third tranchet blow negative	Width of the third tranchet blow negative	Length of the fourth tranchet blow negative	Width of the fourth tranchet blow negative
Jeaninn.	-	62	0	12	6	-	-	-	-	-	-
Jeaninn.	-	71	0	20	5	-	-	-	-	-	-
Jeaninn.	-	72	0	19	5	-	-	-	-	-	-
Jeaninn.	-	73	0	32	9	20	9	-	-	-	-
Jeaninn.	-	74	0	16	9	38	9	-	-	-	-
Jeaninn.	-	75	0	35	16	-	-	-	-	-	-
Jeaninn.	-	76	0	32	24	-	-	-	-	-	-
Jeaninn.	-	77	0	21	20	9	7	12	5	-	-
Jeaninn.	-	92	0	16	4	-	-	-	-	-	-
81.21.1.	-	107	0	12	3	-	-	-	-	-	-
81.21.1.	-	109	0	16	5	-	-	-	-	-	-
81.21.1.	-	135	0	16	5	-	-	-	-	-	-
81.21.1.	-	137	0	18	13	17	12	-	-	-	-
81.21.1.	-	147	0	23.3	3.4	21.8	13.8	-	-	-	-
CA 27	-	125	0	24	14	24	10	-	-	-	-
CA 27	-	126	0	17	8	-	-	-	-	-	-
CA 27	-	146	0	13	4	-	-	-	-	-	-
CA 27	-	171	0	30	17	25	12	17	8	11.7	12.2
-	192-099	275	0	12	13	12	5	13	2	-	-
-	200-102	34	6	17	8	-	-	-	-	-	-
-	200-102	39	4	12	7	7	4	-	-	-	-
-	201-105	1	19	19	9	11	5	-	-	-	-
-	204-103	4	1	14	9	10	10	-	-	-	-
-	204-104	1	4	15	8	8	10	-	-	-	-
-	204-104	1	6	15	10	10	5	-	-	-	-
-	204-104	10	2	15	9	11	6	9	5	-	-
-	214-112	1	14	55	11	-	-	-	-	-	-
-	214-112	2	3	21	18	-	-	-	-	-	-
-	204-102	18	8	9	5	-	-	-	-	-	-
-	204-102	23	3	7.3	8.9	-	-	-	-	-	-
-	204-102	25	2	17	13	19	8	-	-	-	-
-	204-102	25	3	24	12	-	-	-	-	-	-
-	204-102	44	15	11	12	30	13	7	12	-	-
-	204-102	48	4	10	5	11	6	17	13	-	-
-	204-102	75	1	26	12	23	12	-	-	-	-
-	205-102	216	0	16	10.2	-	-	-	-	-	-
-	205-102	330	0	15	7	25	9	-	-	-	-
-	205-102	430	0	11	11	-	-	-	-	-	-
-	205-102	469	0	24	20	-	-	-	-	-	-
-	205-102	539	0	-	-	-	-	-	-	-	-
-	205-102	615	2	19.5	9.1	6.2	4.9	-	-	-	-
-	205-102	763	0	27	12	-	-	-	-	-	-
-	205-102	993	0	9	8	-	-	-	-	-	-
-	205-102	1001	0	22.3	6	-	-	-	-	-	-

Measurements in mm
