# Supplementary material for: The performance of tranchet blows at the Late Middle Paleolithic site of Grotte de la Verpillière I (Saône-et-Loire, France)
Source: PLoS One. 2017 Nov 30;12(11):e0188990. doi: 10.1371/journal.pone.0188990 (PMC5708829; doi:10.1371/journal.pone.0188990)
Supplement: S6 Table — (PDF) [file pone.0188990.s006.pdf]

S6 Table. Length of active edge, bow, back and base of Keilmesser with tranchet blow from Grotte de la Verpillière I.

Inventory number (ancient finds)	Square meter (Floss excavation)	Find number (ID)	Sub find number (Suffix)	Total length of the active edge	Length of active edge 1	Length of active edge 2	Length of the bow	Length of the back	Length of the base
Jeaninn.	-	62	0	25.4	11.5	13.9	12	34.9	11.7
Jeaninn.	-	71	0	44.3	25.4	18.9	30.7	45.2	34.3
Jeaninn.	-	72	0	51	28.1	22.9	25	28.2	22.7
Jeaninn.	-	73	0	46.1	30.1	16	14.3	42.2	28
Jeaninn.	-	74	0	52.3	33.1	19.2	39.3	38	16.9
Jeaninn.	-	75	0	42.2	24.1	18.1	18.1	39.5	35.7
Jeaninn.	-	76	0	44.7	27.3	17.4	33.6	45	39.2
Jeaninn.	-	77	0	38.9	12.1	26.8	22.8	48.8	22.9
Jeaninn.	-	92	0	30.2	16.8	13.4	21.6	33.2	16.3
81.21.1.	-	107	0	38.6	18.5	20.1	30.7	26.1	27.5
81.21.1.	-	109	0	88.9	16.1	72.8	25.6	76.2	21.3
81.21.1.	-	135	0	31.3	16.9	14.4	28.1	30.9	23.2
81.21.1.	-	137	0	59.5	20.8	38.7	34.2	51.5	43.3
81.21.1.	-	147	0	78.6	35	43.6	34.6	61.9	22.8
CA 27	-	125	0	45.8	26.3	19.5	27.6	30.6	30.7
CA 27	-	126	0	37.4	18.3	19.1	33.3	45.7	37.2
CA 27	-	146	0	42.9	27.1	15.8	18.5	31.7	30.5
CA 27	-	171	0	43	21.5	21.5	34.3	44.5	42.6
-	192-099	275	0	27.2	12.4	14.8	19.9	21.7	21
-	200-102	34	6	34.6	16.1	18.5	22.5	35.7	15
-	200-102	39	4	35.4	13.5	21.9	23.4	27.9	23.3
-	201-105	1	19	37.2	19.1	18.1	23	63.8	22
-	204-103	4	1	33.4	14.2	19.2	9.1	34.6	17.5
-	204-104	1	4	27.6	15.3	12.3	14.7	23.2	21.5
-	204-104	1	6	31.8	7.7	24.1	15.1	25	21.1
-	204-104	10	2	30.6	9.8	20.8	23.3	28.1	17.3
-	214-112	1	14	63.2	19	44.2	27.3	56.6	23.7
-	214-112	2	3	62.1	-	-	19	40.9	22
-	204-102	18	8	22	7.5	14.5	13.5	28.9	21.2
-	204-102	23	3	25	8	17	18	22	22
-	204-102	25	2	40	23.2	12.2	25.2	40.4	18.6
-	204-102	25	3	40.1	18.5	21.6	26.3	34.5	35.1
-	204-102	44	15	46.7	16.5	30.2	20.5	40.5	40.5
-	204-102	48	4	42	10.2	31.8	9.2	33	24.2
-	204-102	75	1	48	23	25	34	46.2	30.7
-	205-102	216	0	45	33	12	-	25	24
-	205-102	330	0	43.8	21.8	22	21.8	50	15
-	205-102	430	0	40.9	23	17.9	21.3	44.6	23.1
-	205-102	469	0	38.8	20.4	18.4	6.5	43	34.2
-	205-102	539	0	-	-	-	-	-	-
-	205-102	615	2	28	15.5	12.5	12.7	28.7	14
-	205-102	763	0	35.2	18.4	16.8	33.3	44.3	38.8
-	205-102	993	0	30.5	13.4	17.1	16.9	34.8	21.1
-	205-102	1001	0	28	23	5	26.8	13.8	8.7

Measurements in mm.
