# Supplementary material for: The performance of tranchet blows at the Late Middle Paleolithic site of Grotte de la Verpillière I (Saône-et-Loire, France)
Source: PLoS One. 2017 Nov 30;12(11):e0188990. doi: 10.1371/journal.pone.0188990 (PMC5708829; doi:10.1371/journal.pone.0188990)

S7 Figure. Length and width comparison of Keilmesser with tranchet blow at VP I with Keilmesser at Buhlen (data for Buhlen from Jöris 2001 [8])

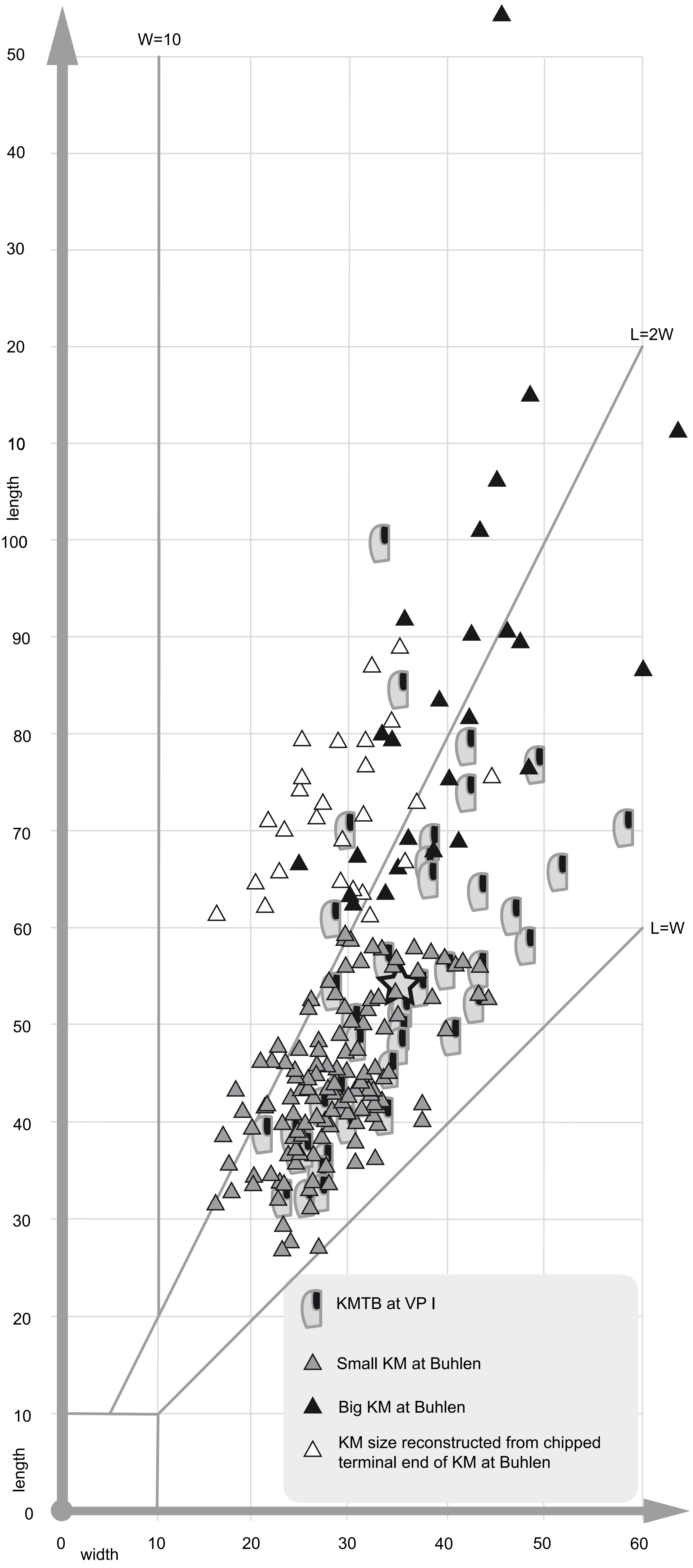

Supplement: S1 Fig — Data of the Buhlen site from Jöris (2001). (PDF) [file pone.0188990.s007.pdf]
